# Adult age differences in the integration of values for self and other

**DOI:** 10.1038/s41598-025-96656-6

**Published:** 2025-04-14

**Authors:** Lena Pollerhoff, Anne Saulin, Marcel Kurtz, Julia Stietz, Xue-Rui Peng, Grit Hein, Anita Tusche, Philipp Kanske, Shu-Chen Li, Andrea M.F. Reiter

**Affiliations:** 1 Lifespan Developmental Neuroscience, Faculty of Psychology, Technische Universität Dresden, Dresden, Germany; 2 Department of Child and Adolescent Psychiatry, Psychosomatics and Psychotherapy, University Hospital Würzburg, Würzburg, Germany; 3 Translational Social Neuroscience Unit, Department of Psychiatry, Psychosomatics, and Psychotherapy, University Hospital Würzburg, Würzburg, Germany; 4 Clinical Psychology and Behavioral Neuroscience, Faculty of Psychology, Technische Universität Dresden, Dresden, Germany; 5 Department of Psychology, Queen’s University, Kingston, ON Canada; 6 Center for Neuroscience Studies, Queen’s University, Kingston, ON Canada; 7 Max Planck Institute for Human Cognitive and Brain Sciences, Leipzig, Germany; 8 Centre for Tactile Internet with Human-in-the-Loop, Technische Universität Dresden, Dresden, Germany; 9 German Centre of Prevention Research on Mental Health, Julius-Maximilians-Universität Würzburg, Würzburg, Germany; 10 Department of Psychology, Julius-Maximilians-Universität Würzburg, Würzburg, Germany; 11 Adaptive Learning Psychology and Neuroscience Lab, Center for Human Brain Health, University of Birmingham, Birmingham, UK

**Keywords:** Prosocial behavior, Cognitive functioning, Inhibitory control, Adult lifespan, Drift-diffusion modeling, Psychology, Human behaviour

## Abstract

Previous research suggests that older adults may display more prosocial behavior than younger adults. However, recent meta-analyses indicate that effects are heterogeneous, may be small, and are influenced by how prosociality is measured. Further, the precise cognitive and computational factors contributing to age-related differences in prosocial behavior remain largely unknown. In this study, we utilized a modified dictator game to combine a value-based decision framework with Bayesian hierarchical drift-diffusion modeling to investigate prosocial decision-making in a sample of younger (*n* = 63) and older adults (*n* = 48). We observed differences in how older and younger individuals incorporate information corresponding to potential gains for themselves (self) and another person (other) to reach a (potentially prosocial) decision. Younger adults integrated values for benefits for themselves and others in the decision-making process and demonstrated increased decision-making efficiency by effectively integrating both sources of information. In contrast, older adults showed improved decision-making efficiency when solely considering values for self and others separately. Interestingly, individual differences in the capacity of inhibitory control in older adults moderated the observed age effects: older adults with stronger inhibitory control abilities made decisions based on the integrated information of benefits for themselves and others. Together, these findings offer new insights into the behavioral and computational mechanisms influencing age effects in prosocial decision-making.

**Supplementary Information:**

The online version contains supplementary material available at 10.1038/s41598-025-96656-6.

## Introduction

In 2050, the global population will comprise an equal number of older and younger individuals and thus, aging global populations present significant health, social, and economic changes in the coming decades^1^. This change is linked to challenges as well as opportunities. Prosocial behavior, for example, is a vital component for the functioning of societies^2^ and has a profound impact on individuals’ mental and physical well-being across their lifespan^3–6^. Past research has provided evidence for higher prosociality in older as compared to younger adults^7–10^. However, recent meta-analyses indicated that this effect might be small. Moreover, these meta-analyses have revealed significant heterogeneity in the association of adult age and prosociality, depending on how prosociality is measured^9–11^. Importantly, cognitive mechanisms underlying age-related differences in prosocial behavior, as well as factors moderating them, remain yet to be identified^12^.

Prosociality incorporates various behaviors such as cooperating, helping, or sharing^13,14^, all of which involve a cost to the self in order to benefit others^15,16^. Thus, a decision to engage in prosocial acts can be cast as the result of a cost-benefit calculation based on integrating values for oneself and others^12,17,18^. Interestingly, studies in the non-social domain suggest differences between younger and older adults in tasks that require integrating information from different sources or cost-benefit trade-offs^11,19–21^, potentially due to aging-related changes in available cognitive (control) resources^21,22^. A decline in cognitive resources in older age might affect the integration of all available information relevant to the decision-making process^23^ and computationally, lower cognitive abilities have been related to lower efficiency in the decision process^24,25^. In line with this notion, it has been proposed that older adults may rely on processes that require lower cognitive demands during decision-making, aiming to minimize cognitive effort^26,27^, for example, by analyzing and/or integrating less information^28–30^.

To explicitly test the factors underlying potential age-related differences in (pro)social decision-making, drift-diffusion models^31,32^ are a promising tool to computationally describe trade-offs like the decision to help someone at one’s own cost^33–36^. That is, drift-diffusion models provide a quantitative framework to delineate cognitive subcomponents involved in cost-benefit calculations by leveraging the combined information of response time and choice outcome. In detail, they assume that a noisy signal representing the relative value of (most often) two options, like helping someone or not, is integrated at each moment, and a decision is made when the accumulated evidence reaches the boundary of the corresponding choice option^37–39^. This process can be characterized in terms of three parameters that each capture a different aspect of the decision process: Firstly, the *drift rate*, a parameter which quantifies the efficiency of the decision process. The larger this parameter, the more efficiently the decision is made. Secondly, the *initial bias*, a parameter which is a marker for a participant’s a-priori bias towards one decision option before accumulation of evidence has started. This reflects that if one of the two response options (like helping someone or not) has a higher expected value for an individual, participants shift the starting point towards this favored option. And thirdly, the *boundary separation* which is a measure for how much evidence needs to be accumulated to reach a decision. The larger this parameter, the larger a participant’s response caution favoring accuracy over speed^32^. In the field of psychology, drift-diffusion models were initially applied to reveal the underlying mechanisms in the realm of perceptual decision-making^40–42^. Since then, they have been successfully applied in a wide range of decision-making paradigms^43–45^ and contributed to the better understanding of previously conflicting results in experimental economics^46^. Drift-diffusion models have also helped shed light on social processes such as stereotyping^47^, age-related changes in gaze-processing^48^ and even collective decision-making^49^. In particular, past work has demonstrated that drift-diffusion models can be successfully applied to delineate cognitive subcomponents that drive decisions in the realm of (pro)social decision-making^18,50–53^ and how we make decisions for ourselves in comparison to how we make decisions for others^54^. Thus, this approach is a valuable tool to gain insights into the cognitive mechanisms underlying differences in prosocial decision-making between younger and older adults^55^.

Previous studies leveraging drift-diffusion models to study age-related differences in decision-making observed lower drift rates when the task required the integration of different information, i.e., a reduced decision-making efficiency in older but not younger adults when combining different sources of information^56^. More generally, older adults exhibited lower drift rates in the perceptual and memory domain^57,58^. Moreover, on a range of non-social tasks, older adults task-independently adopted higher boundary separations as compared to younger adults (i.e., they applied more cautious response criteria than younger adults^58^).

Building on these findings in the non-social domain, we aimed to provide insights into the precise mechanisms underlying age effects in the cost-benefit tradeoff of social decision-making. More specifically, we asked whether older and younger adults differ when integrating values for themselves and others to reach a social decision and whether we can relate these differences to interpretable cognitive subcomponents like decision-making efficiency, evidence accumulation, and initital response bias for self-serving or other-serving options. To test this, younger and older adults performed a widely used task to measure prosociality (modified dictator game^18^). In this paradigm, the decision-maker is exposed to a set of choice options with varying payoffs for oneself and another person, which can be integrated or considered independently to make a decision. This allowed us to examine age group differences in value-integration processes during prosocial decision-making by modeling their choice behavior and reaction times using state of the art drift-diffusion modeling^59,60^.

Further, we were interested in factors that might influence the cost-benefit tradeoff of prosocial decision-making, and age differences therein. In general, there is increasing evidence that socio-emotional and socio-cognitive capacities like empathy, compassion, and Theory of Mind predict prosociality^61–70^. However, studies testing whether the influence of empathy and Theory of Mind on prosocial behavior differs as a function of age showed mixed results^71–76^. Moreover, general cognitive abilities have been suggested to play a moderating role in age-related differences in prosocial behavior^12^. For instance, older age is associated with reduced inhibition and cognitive functioning per se^77–80^, both of which are negatively associated with giving behavior in adult age^81–88^. To investigate the role of such potential social and cognitive moderators of age differences in social decision-making, we additionally assessed social, general cognitive and inhibition capacity in our participants.

Based on previous studies in the non-social domain, we hypothesized that older adults use the integrated information about their own and the other person’s payoff to a lesser extent than younger adults. This would be reflected in a smaller interaction effect of self and other payoff in older as compared to younger adults. Furthermore, we hypothesized that age-related differences in prosocial decision-making may be attributed to the efficiency of the prosocial decision-making process (i.e., different drift rates in older adults than younger adults, especially for other-serving decisions), and/or the initial bias towards other-serving decisions (i.e., different initial bias in older adults than younger adults). Because response caution is commonly increased for older adults (i.e., larger boundary separation in older adults than younger adults in line with Salthouse’s cognitive slowing hypothesis^89^), we additionally accounted for these age-related differences in the boundary separation. Lastly, we explored to what extent individual differences in cognitive and social abilities may modulate these potential age-related differences in decision-making efficiency and initial bias towards other-serving decisions.

## Methods

The current study was part of a bigger project consisting of three testing sessions. Here, we focused on the behavioral data from a social choice task (a modified dictator game^18^), the EmpaToM task^90^, a paradigm to assess socio-affective and socio-cognitive processes, and a cognitive functioning battery. In a separate session, participants completed an inhibitory control battery which we include as a potential moderator of our task effects^91^. Other data gathered within this larger project have been (or will be) reported elsewhere^92^.

### Participants

A total of 126 participants participated in the relevant test sessions of the study: 70 younger adults, age range: 18–30 years, and 56 older adults, age range: 65–78 years. All participants were recruited via flyers and newspaper announcements in the greater Dresden city region as well as the database of the Lifespan Developmental Neuroscience Lab at Technische Universität Dresden. Older adults were additionally recruited from sports, language and university courses as well as local choirs. Sample size estimation was mainly based on the work by Hutcherson and colleagues who tested 66 participants and analyzed 51 data sets using a one-group design^18^. We thus aimed for a comparable number of participants per age group. A post-hoc sensitivity analysis using G*Power 3.1 indicated that given α = 5% and considering the most complex regression model (age group * €self * €other), the sample size had 80% power to detect a true effect with an effect size of f ≥ 0.08 (F ≥ 2.95).

The present sample largely overlapped with a previously published study on a different experimental task completed within the same sessions^92^. We did not observe significant age group differences concerning years of education, relationship status, or residence. The final sample comprised 63 younger adults and 48 older adults. For recruitment, exclusion criteria and sample characteristics of the final sample please refer to the supplementary material (pp. 1, Table S1).

## Materials and procedure

Participants provided written informed consent before the sessions. Participants filled in a questionnaire on socio-demographic characteristics (see Table S1). They were compensated with 8.50€ per hour for their participation and a bonus of a maximum of 11€ based on their decisions in the social choice task. Ethical approval was granted by the Technische Universität Dresden ethics committee in accordance with the Helsinki declaration (EK 486 112 015).

## Social choice task: modified dictator game

**Fig. 1:**
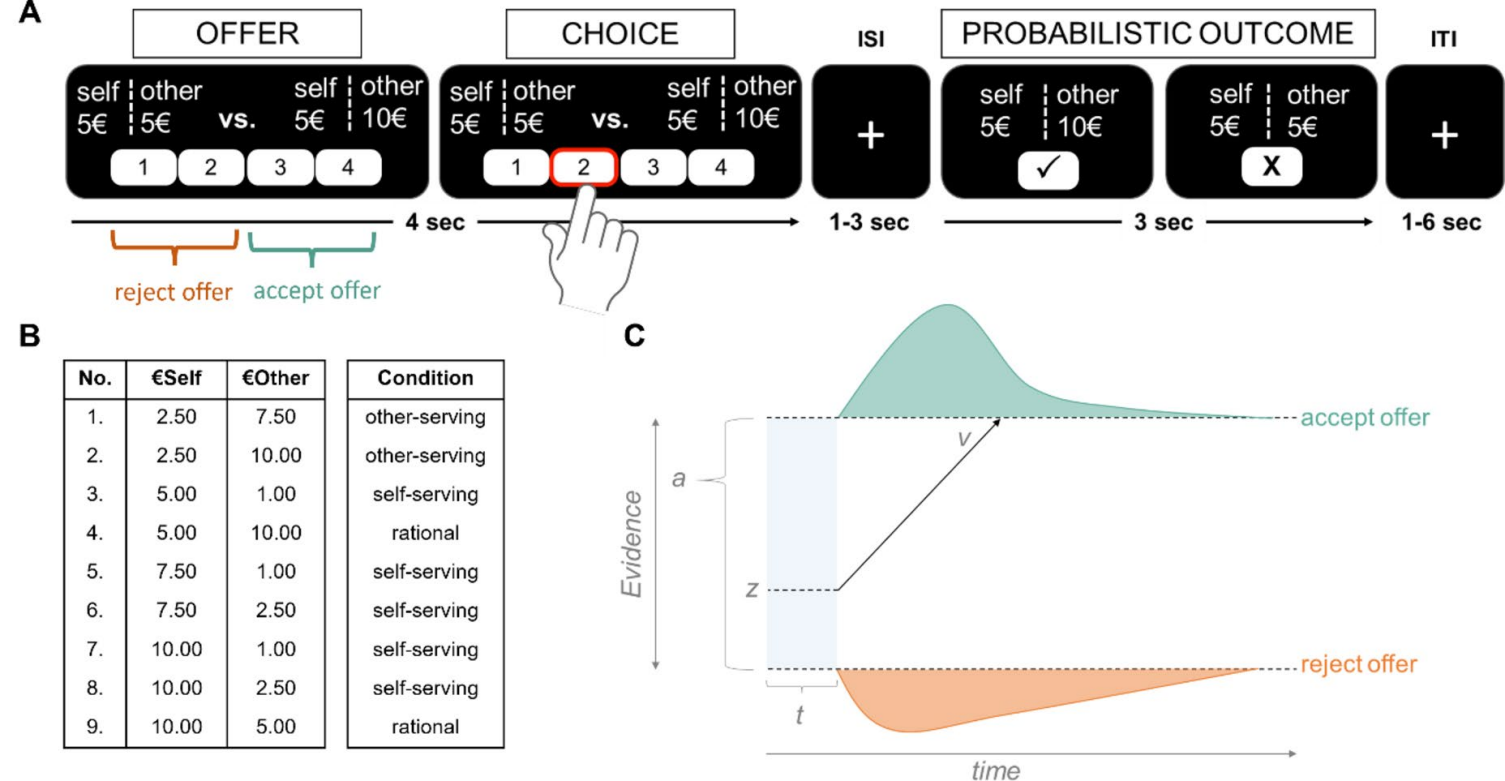
Modified dictator game to assess the integration of values for self and others and schematic illustration of the drift-diffusion model. Note. (**A**) Illustration of the social choice task (modified dictator game based on^18^). In each trial, participants accepted or rejected a varying monetary offer that affected payoffs for themselves (self) and another player (other) versus a constant default distribution of money (5€ for both players) using a 4-point scale (1 = strong no, 2 = weak no, 3 = weak yes, 4 = strong yes - toward the varying monetary offer; scale direction counterbalanced across participants). Outcomes were implemented in a probabilistic manner such that choices were implemented in 80% of trials or reversed in 20% of trials. (**B**) Nine proposed offer types, representing different monetary distributions between the participant (€self) and its partner (€other) and post-hoc classification of the offer types into self-serving, other-serving, and rational condition. Every offer type was shown 20 times (using a random jitter of up to 0.40€ to reduce habituation), randomly intermixed across the task. (**C**) The drift-diffusion model is used to conceptualize latent variables underlying the decision process, which are defined by the noisy accumulation of information (squiggly line). The non-decision time (ndt) controls for both sensory and motor-related processes. The v-parameter (drift rate) represents the speed of the accumulation process. The a-parameter (boundary separation) describes the distance between the two boundaries. The z-parameter indicates the initial bias of the accumulation process with respect to the two boundaries. A decision is made the moment one of the two boundaries is crossed (i.e., enough evidence accumulated). In the example trial (Fig. 1A), choosing “1” or “2” would correspond to rejecting the offer (lower boundary), whereas choosing “3” or “4” would correspond to accepting the offer (upper boundary). Upper boundary = accepting the offer (i.e., rejecting the default distribution), lower boundary = rejecting the offer (i.e., accepting the default distribution).

Two participants of the same age group and self-reported gender completed a social choice task (modified dictator game^18^). To emphasize the social context, participants were first asked to consider the other participant (whom they saw at the beginning of the session) as their partner in the following game (see complete instructions in the Supplement pages 60 ff). Next, each participant completed nine practice trials of the computerized task to ensure task comprehension, followed by the 180 trials of the main dictator game. In each trial, participants chose between a proposed offer with varying monetary distributions for themselves (“€self” value) and their partner (“€other” value), or a constant default distribution of money (5€ for both) (see Fig. 1A). The location of the two offers on the screen (left vs. right) was randomized across participants. The proposed offer varied from 1€ to 10€ and was drawn from one of the nine offer types shown in Fig. 1B. Every offer type was shown 20 times, randomly intermixed across the experiment. As in Hutcherson et al. (2015)^18^, to avoid habituation and repetition effects, the values of €self and €other were randomly jittered by plus or minus 0–0.40 € (10€ were always jittered downward). Participants had up to four seconds to choose between the default distribution and the proposed offer on a four-point scale of “strong yes” (accepting the varying proposal) to “strong no” (rejecting the proposed offer in favor of the default). Failures to respond within four seconds yielded the automatic acceptance of the default and the on-screen feedback “too slow.” To reduce social desirability effects, we further implemented the chosen options probabilistically (as in^18^): with an 80% probability the chosen option was logged after the trial, while the non-chosen option was implemented in the remaining 20% of the time. Participants were explicitly informed about this probabilistic aspect of the task in the instructions. Thus, in the case of observing a self-serving choice, a partner could not be certain of whether this was indeed a selfish choice by the player, or a probabilistic outcome implemented by the computer algorithm. Participants were informed that at the end of the task, one trial would be randomly chosen and implemented (determining the bonus pay at the end of the session).

## Inhibitory control battery

To investigate whether inhibitory control moderates the relationship between adult age and the way values for oneself and others are integrated, we used a previously validated, computerized task battery designed to assess (amongst other constructs) inhibition performance (adopted from^91^). The three inhibition tasks included a Go-Nogo, Stroop, and Stop Signal task (see Table 1 for details). Participants were instructed to react as fast an accurately as possible in all tasks. For the Go-Nogo and Stroop tasks, we calculated inverse efficacy scores (IESs^93^). For the Stop Signal task, we computed the stop signal reaction time (SSRT) using the quantile method^94^. On each trial of the Stop Signal task, a fixation cross (750 ms) was followed by an arrow pointing to either the left or the right. There were 200 “go” and 40 “stop” trials. On “go” trials, the arrow was shown for 1,000 ms, and participants were instructed to press either the left or the right response key (“Y” or “M”) according to the direction of the arrow. On “stop” trials, the sideward-pointing arrow was replaced by an upward-pointing arrow after a variable stop-signal delay (SSD), and participants had to withhold the response. To achieve a stop-trial error rate of approximately 50% for all participants, the initial stop-signal delay of 200 ms was adapted after each “stop” trial by adding 50 ms for a (correct) nonresponse and subtracting 50 ms for a (faulty) response. Randomization of trials was constrained so that “stop” trials were separated by at least five “go” trials.

**Table 1:** *Inhibition Tasks*. *(adapted from* ^91^ *)*

Task (adapted from)	Task description	Outcome measure
Go-Nogo	Two vertically or horizontally arranged dots (500ms) were presented. In “go” trials (vertically arranged dots), participants should press the response key. In “nogo” trials (horizontally arranged dots), participants should not react. In total the task consisted of 280 “go” and 40 “nogo” trials. At least five “go” trials were in between a “nogo” trial.	IES^1^ (average RTs for correct “go” trials are divided by the proportion of correct “nogo” trials)
Stroop ^55,66^	A stimulus was presented (1000ms) illustrating a row of either one, two, three, or four identical digits ranging from 1 to 4. The number and denotations of the digit could either be congruent (80 trials) or incongruent (80 trials). Instruction indicated to ignore denotations but respond in accordance with the number of presented digits by pressing one of four corresponding response keys.	IES^2^-difference (IES from congruent vs. IES from incongruent trials)
Stop Signal ^94,97^	Participants saw an arrow that either pointed to the left or right side. In total, 200 “go” and 40 “stop” trials were included. In “go” trials (arrow presented for 1000ms), participants should press the left or right response key corresponding to the direction of the arrow. In “stop” trials (upward-pointing instead of sideward-pointing arrow), participants were instructed to withhold the response after a variable stop-signal delay (SSD). At least five “go” trials were presented between “stop” trials.	SSRT (quantile method^3^ based on^94^

Note. Each trial starts with a fixation cross (750ms). IES = inverse efficiency score; RT = reaction time; SSRT = stop signal reaction time.

^1^IES, derivded by dividing the average RT for accurate “go” trials by the ratio of correct response in “nogo” trials (indicated in milliseconds).

^2^First, IES were calculate separately for congruent and incongruent trials by dividing the corresponding average RT by the corresponding accuracy rate. After that, the difference between IES in congruent and incongruent trials was calculated for each participant.

^3^RTs from correct go trials are sorted in ascending order, and quantiles of RTs related to error rates on “stop” trials are selected. The mean SSD is then subtracted from the quantile RT to calculate SSRT (see also^91,94^).

Z-scores of all task measures were used to build a composite inhibition score for each participant by calculating the mean of these z-scores. Z-scores were estimated based on the following approach: For models including the whole sample, z-scores were calculated based on the whole sample (younger and older adults) to reflect differences between younger and older adults. For models including only one age group, z-scores based on the specific age group were calculated to reflect intra-group differences in one of the age groups. See Table S1 for descriptive and inferential statistics of task-specific and composite inhibition scores.

## Cognitive functioning battery (fluid vs. verbal abilities)

In order to be able to test for a moderating role of general cognitive abilities in age-related differences in prosocial behavior^12^, we characterized differences between younger and older adults in cognitive functioning by adopting a previously used task battery^98^ that included the following tests: Trail Making tests A and B (TMT A and B^99^), the Identical Pictures test (IDP^100^), the Digit Span backward (DSb^101^), and the Spot A Word test (SAW^102^). Matching the procedure of^98^ and^92^, mean composite scores were calculated based on z-scored test values to derive measures of fluid and verbal abilities. As in the inhibition task battery, two different approaches were followed for calculating z-scores and corresponding composite scores: For models including the whole sample, z-scores were estimated based on the entire sample. For models including only one age group, z-scores were calculated with the values of this specific age group. To derive the average composite of fluid abilities, we assessed processing speed with the Identical Picture Test, working memory with the Digit Span backward, visual attention with the Trail Making Test A, and complex attention with the Trail Making Test B. Spot A Word test scores (performance and RT), were used for the average composite score of verbal capacities. Previous findings from a larger population-based lifespan sample^103^ suggested an age-related decline in fluid capacities, but an experience-related increase in crystallized abilities in older adults compared to younger adults. In line with those findings^103^, younger adults (YA) showed better cognitive abilities concerning our composite score of fluid abilities compared to older adults (OA) (YA: *M* = 0.42, OA: *M* = -0.57, *t* = 10.26, *p* < 0.01), whereas older adults outperformed younger regarding the composite score of verbal abilities as assessed with the Spot A Word test (OA: *M* = 0.26, YA: *M* = -0.20, *t* = 4.52, *p* < 0.01). Descriptive and interferential statistics for the different cognitive tasks and composite scores can be found in Table S1.

## EmpaToM paradigm (empathy, compassion, and theory of mind)

The EmpaToM^90,104^, a video-based social understanding task, was designed to measure empathy, compassion, and Theory of Mind (ToM) within the same individual and task. Every trial consisted of a ~ 15-second video clip in which a male or female narrator reported an autobiographic experience whose content could be either neutral or negatively emotional. Afterward, participants indicated on two different rating scales: (i) how they felt after the specific video, that is, the current valence of their emotion (empathy measure), and (ii) how much compassion they felt for the individual in the video (compassion measure). Last, participants responded to a multiple-choice question, which either required ToM (“[name of the narrator] thinks that.”) or factual reasoning (“It is correct that…”) as a control measure. Matching previous studies^92,98,105^, the variables were operationalized as follows: (i) behavioral empathy was defined by mean valence ratings in emotional minus neutral trials, (ii) behavioral compassion was operationalized by mean compassion ratings across emotional and neutral trials and (iii) behavioral ToM was analyzed by utilizing the accuracy in ToM-trials. For further details, see^92^.

## Statistical analyses

Statistical analyses were performed using Matlab (2022b; MATLAB, 2020), R (Version 4.1.2; R Core Team, 2018) with RStudio, and Python (Version 3.6.13). Age differences concerning sample characteristics, inhibition, cognitive functioning, empathy, compassion, and ToM (see Table S1) were examined with a non-parametric robust yuen test^106^ or *χ*-test^2^. If the data fulfilled the assumptions of homoscedasticity and normality, an independent t-test was used (indicated by an asterisk in Table S1). The two primary outcome variables of interest for the dictator game were choice behavior and reaction time (RT). RTs were analyzed for responses given on all 180 choice trials. Rare trials with RTs < 200ms were removed (*M* = 1.91, *SD* = 3.13 of removed trials across all participants). In the following, RTs are indicated in seconds.

To model the effect of the different offer types as defined by our experiment, trials showing the different offers (Fig. 1B) were classified into a condition variable (self-serving, other-serving, and rational condition). Trials in the other-serving condition included offer types for which acceptance yielded costs to participants to benefit their partner (compared to the default option of 5€ for both players). In other words, an offer was considered other-serving with €self ≤ 5€ but €other > 5€. The self-serving condition mirrored the other-serving condition. Specifically, offers were classified as self-serving if accepting this offer benefitted the participant at a cost to their partner (i.e., accepting offers with €self ≥ 5€ but €other < 5€). Two offer types (accepting €self5:€other10 and €self10:€other5) were categorized as rational condition as accepting these offers would never result in less money for the participant or the other as compared to the standard offer. Thus, their acceptance can neither be classified as prosocial nor selfish behavior but would be the rational choice as it is always advantageous for one of the parties without a cost to the respective other. Refer to Fig. 1B for the classification of the nine different offer types into the condition variable. This classification allowed us to focus on the decision process in the other-serving condition as defined by prior studies on altruistic choice^18,52^.

See the paragraphs below for detailed information on the analyses of choice behavior, RTs and modeling. All scripts for statistical analysis are available via: https://osf.io/zu4p3/?view_only=8b5cf6703f3b4d04a7122308c31445f4.

.

## Mixed model regression analyses of choice behavior and RTs in the DG

We ran two separate linear mixed-effects models to examine age effects regarding the trial-by-trial choices and RTs in the DG, using the *mixed* function from the “afex” package (Version 1.1.-0) in R^107^. For all mixed models, the *F* test statistic and *p*-values based on the Satterthwaite approximation are reported. When simple slopes were extracted from the mixed models, 95% confidence intervals were calculated using the *emtrends* function from the “emmeans” package (Version 1.7.3) in R^108^. Effect sizes d were calculated based on the recommendations in^109^.

Choice behavior (as derived from ratings on the four-point Likert scale) was included as a continuous dependent variable ranging from 1 = *strong no* to 4 = *strong yes* such that higher values indicated a stronger preference for the proposed offer compared to the default distribution (5€ per person). RTs were also treated as a continuous variable. In both models, age group, amount distributed to oneself (€self), and amount distributed to the other person (€other) were included as fixed effects. In addition to the main effects, we also tested for 2-way and 3-way interactions. To account for potential time-related effects, we included a continuous trial variable (1 to 180) as a covariate in the fixed effects. Before fitting the random effects (including random intercept and random slopes), a model with and without the time covariate was compared via the loglikelihood ratio test (R function *anova*), and the model with the lower AIC was selected. Based on a conservative approach, the maximal random effect structure was fitted in the next step^110^: Participant ID was included as a random intercept; the model also allowed for random slopes for the interaction of €self × €other and correlations among the random effect parameters. Whenever convergence warnings occurred, the number of iterations was increased, and the optimizer was changed to “bobyqa” for the respective model space.

Moderation analyses concerning inhibitory control, a composite of fluid abilities, and a composite of verbal abilities, as well as empathy, compassion, and ToM were further investigated in separate post-hoc models, separately for choice behavior and RTs. In separate models for younger and older adults we tested whether trial-by-trial choice behavior and RTs were predicted by a 3-way interaction of the moderator variable with €self × €other (e.g., whether the interaction term of inhibition × €self × €other predicted choice behavior in older adults). Thus, a moderation effect was defined by a significant 3-way interaction either predicting choice behavior or RTs in younger adults or older adults. We adjusted the p-values derived from models including the composite scores of fluid an verbal abilities (derived from the same cognition battery), as well as p-values derived from the models including empathy, compassion, and ToM (derived from one task, the EmpaToM task), as moderators to control for the false-discovery rate (FDR, *p-adjust* function from the “stats” package in R, Version 4.1.2^111^).

### Hierarchical drift-diffusion modeling

Bayesian hierarchical drift-diffusion modeling (HDDM^59,112^) was applied to model the choice and RT data from the modified dictator game. HDDM allows for the exploitation of between- and within-subject variability in relatively small sample sizes with the help of Bayesian estimation methods. Diffusion modeling of choice and RT data was carried out with the Python package HDDM (Version 0.8.0^112^).

For the HDDM analysis, binary outcomes were used that were computed based on participants’ choice behavior on the four-point Likert scale (as measured in the dictator game). To achieve this, we recoded choices into a binary outcome format (accept proposed offer vs. reject proposed offer) following previous work^18,52,113^. A participant’s choice of “strong no” or “weak no” indicated the rejection of the proposed offer (and acceptance of the default distribution) and was coded as zero. Choosing “weak yes” or “strong yes” defined the acceptance of the proposed offer, coded as one.

The HDDM framework uses both trial-by-trial choices and RTs to estimate latent variables characterizing the (prosocial) decision process (*v*, *z*, and *a*-parameters, Fig. 1C). The *v*-parameter (drift rate) captures the speed of the accumulation process, with which the participant decides to accept or reject the offer. Thus, the v-parameter represents the efficiency of the prosocial decision-making process itself^114^. The *z*-parameter describes the initial choice bias with which a participant enters the choice process, i.e., the extent to which a participant favors one choice option over the other (accept vs. reject) before entering the evidence accumulation process^50^. Lastly, the *a*-parameter (boundary separation) reflects the amount of relative evidence to be accumulated to choose one of the two options. A large *a*-parameter indicates more cautious response behavior, whereas a small *a*-parameter indicates response behavior favoring speed over caution^115,116^.

According to the theoretical reasoning of the drift-diffusion model, the integration of potential values associated with €self and €other during the decision process should be reflected by the modulation of the *v*-parameter^35,114^. The categorical effect of offer type, presented by the condition variable (other-serving vs. self-serving vs. rational), should potentially influence the modulation of the *z*-parameter and/or the *a*-parameter. These two parameters reflect the pre-decisional characteristics of the decision-making process. Typically, these parameters are not thought to be modulated by trial-by-trial factors. However, in the present study, the condition sets the stage for the accumulation of the exact point values. Including these modulations allowed us to disentangle potential point value effects and more general biases towards prosocial or selfish decision options. Please note that based on this rationale, we did not test for a general effect of condition on drift-rate. Additionally, it is important to note that the combined information of €self and €other constitutes the condition. Therefore, including the condition as an additional potential regressor on the v-parameter would introduce collinearity and lead to model instability.

How exactly do older and younger adults make decisions in the dictator game? To address this question and test which components of the social decision process – such as concerns for benefits for themselves (€self) or others (€other) – best accounted for the observed choices and RTs, we estimated 12 HDDM regression models separately for each age group (see Table 2 for an overview). HDDM regression models allow for testing within-participant effects on the different DDM parameters which properly accounts for within vs. between subject variances. Model definition thus follows the logic of other regression models including main and interaction effects. Accordingly, HDDM regression model estimation yields regression weights for the effects hypothesized which again are interpreted analogously to regression weights in other types of regressions.

**Table 2:** *Overview of the models estimated (separately for younger adults and older adults) and their deviance information criterion (DIC) values.*

Model	Formula	DIC younger adults	DIC older adults
1	-	28176.61	20887.71
2	v ~ preference	27346.09	20009.52
3	v ~ €self	25771.08	18752.34
4	v ~ €other	26435.66	19104.88
5	v ~ €self + €other	20648.23	17758.97
6	v ~ €self x €other	19497.98	17636.23
7	a ~ condition	27058.21	20728.50
8	z ~ condition	23057.16	19253.28
9	a ~ condition, z ~ condition	22800.55	19099.65
10	v ~ €self x €oher, a ~ condition	19314.51	17535.85
11	v ~ €self x €other, z ~ condition	19181.72	17576.00
**12**	**v ~ €self x €other**,** a ~ condition**,** z ~ condition**	**19066.29**	**17479.19**

*Notes*. The winning model is indicated by bold font. 1 = baseline model, i.e., no modulation by experimental variables, 2 = v is modulated by preference variable (post-hoc-defined variable where the choices of 1 and 4 were classified as strong preference, and 2 and 3 were classified as weaker preference), 3 = v is modulated by €self, 4 = v is modulated by €other, 5 = v is modulated by €self, and additionally varies by €other, 6 = v is modulated by an interaction of €self x €other, 7 = a varies by choice option, 8 = z varies by choice option, 9 = a and z vary by choice option, 10 = v is modulated by an interaction of €self x €other, and a varies by choice option, 11 = v is modulated by an interaction of €self x €other, and z varies by choice option, 12 = v is modulated by an interaction of €self x €other, and a and z vary by choice option.

In the simplest model we tested, all parameters were fixed across conditions (please refer to Fig. 1B for definition of the condition variable). Progressively more complex models included regression weights only for self-regard (€self), only other-regard (€other), or their combination. In the full model, the interaction of values of €self and €other (reflecting the tradeoff between self- and other-regard) modulated the drift rate (*v*), and condition-related changes (other-serving vs. self-serving vs. rational) of the boundary separation (*a*) and initial bias (*z*) were assumed. Considering evidence that older adults behaved more prosocially than younger adults, the other-serving condition was used as the intercept, i.e., reference level, of the regression model. Thus, the intercept of the condition effect on the *a*- and *z*-parameters can be interpreted as the absolute initial bias toward accepting the other-serving offer. In turn, the estimated coefficients of the self-serving and the rational condition can be understood as a decrease or increase in initial bias towards accepting the proposed offer *relative* to the acceptance of the other-serving condition.

Besides the three main parameters, each model included the non-decision time ndt and allowed for trial-by-trial variations of the initial bias (*sz*), the drift rate (*sv*), and the non-decision time (*sndt*) fixed across conditions. As mixed designs (between- and within- subject effects) are currently not implemented in HDDM to a satisfactory degree, we fitted all 12 models separately for younger adults and for older adults as recommended by the authors of the toolbox^117,118^. Thus, after estimating the models separately for younger and older adults, the traces of the respective winning model’s parameters can be extracted and compared. This allows for testing age-related differences despite separate model estimation. Default values for priors and hyperpriors, as provided by HDDM, were used for the estimation process^112^. All models (i.e., the included parameters) were visually inspected for convergence with respect to traces, autocorrelations, and marginal posteriors (see Figures S5 and S6). Due to the visual appearance of convergence problems in the estimation process of the a-parameter (especially with respect to older adults’ choices and RTs), we increased iterations to 4000 and burn-ins to 2000 (for recommendation see^119^. Further, we increased the possibility of potential outliers to 10% to improve model fit (as recommended^120^). This approach satisfyingly improved convergence for nearly all parameters, (see Figures S2). Convergence for the *a*-parameter was still suboptimal, which is why the results with regard to the *a*-parameters should be interpreted with caution.

Model convergence was further checked with the Gelman-Rubin statistic (all values < 1.01^121^). In the next step, posterior predictive checks were performed. In detail, we compared our observed data with 500 simulated datasets based on the posterior of our model^112^. If the empirical data fell within the 95% probability interval of the simulated data, the model was considered to describe our data well. We extracted the parameters of interest (*v*, *z*, and *a*-parameter) from the respective winning model for further analyses. Statistical analyses were performed on the mean group posteriors, and we considered an effect significant if the 95% density interval excluded zero.

Linear models were calculated to see whether the pre-defined moderators (inhibitory control, cognitive functioning, empathy, compassion, and ToM) predicted the mean of the individual posteriors of the drift rate (*v*-parameter), initial bias (*z*-parameter), and boundary separation (*a*-parameter), separately for younger and older adults (function *lm*, from the “stats” package (Version 4.1.2^111^), and *summaryh* function from the “hauselin/hausekeep” package (Version 0.0.0^122^) in R.

### Fehr-Schmidt preference

In addition to estimating parameters that characterize the decision process itself, we estimated individual Fehr-Schmidt preferences^123^. This approach takes into account the trial-by-trial relationship between rewards for the self and rewards for the other in order to characterize an individual’s preference for making prosocial decisions. Thus, the higher this estimated value, the stronger an individual’s prosocial preference.

In order to identify potential converging evidence from the DDM approach and the Fehr-Schmidt model, we tested pairwise spearman correlations between the DDM v-parameter regression weight and the z-parameter regression weights using the R-packages corrplot^124^ and BayesFactor^125^.

All data and materials are publicly available at: https://osf.io/zu4p3/.

## Results

### Generous versus selfish choices

Younger adults chose generously (i.e., accepted an other-serving offer that would benefit the other at the cost of themselves) in 17% of the other-serving condition trials and chose selfishly (i.e. accepting an self-serving offer at the cost of the partner) in 33% of the self-serving condition trials. Older adults made generous decisions in 25% of the other-serving condition trials and opted for selfish choices in 38% of the self-serving condition trials. There were no significant age group differences in accepting self-serving versus other-serving offers (age group × condition interaction: *F*(15261.09) = 0.02, *p* = 0.89). However, we found age group differences such that older adults generally favoured offers (compared to the constant default distribution of 5€ per person), independent of if the offer was self-serving or other-serving (main effect age group: *F*(108.97) = 4.91, *p* = 0.03).

### Age group differences in integrating values for oneself and other during social decision-making

#### Choice behavior as a value integration process

**Fig. 2:**
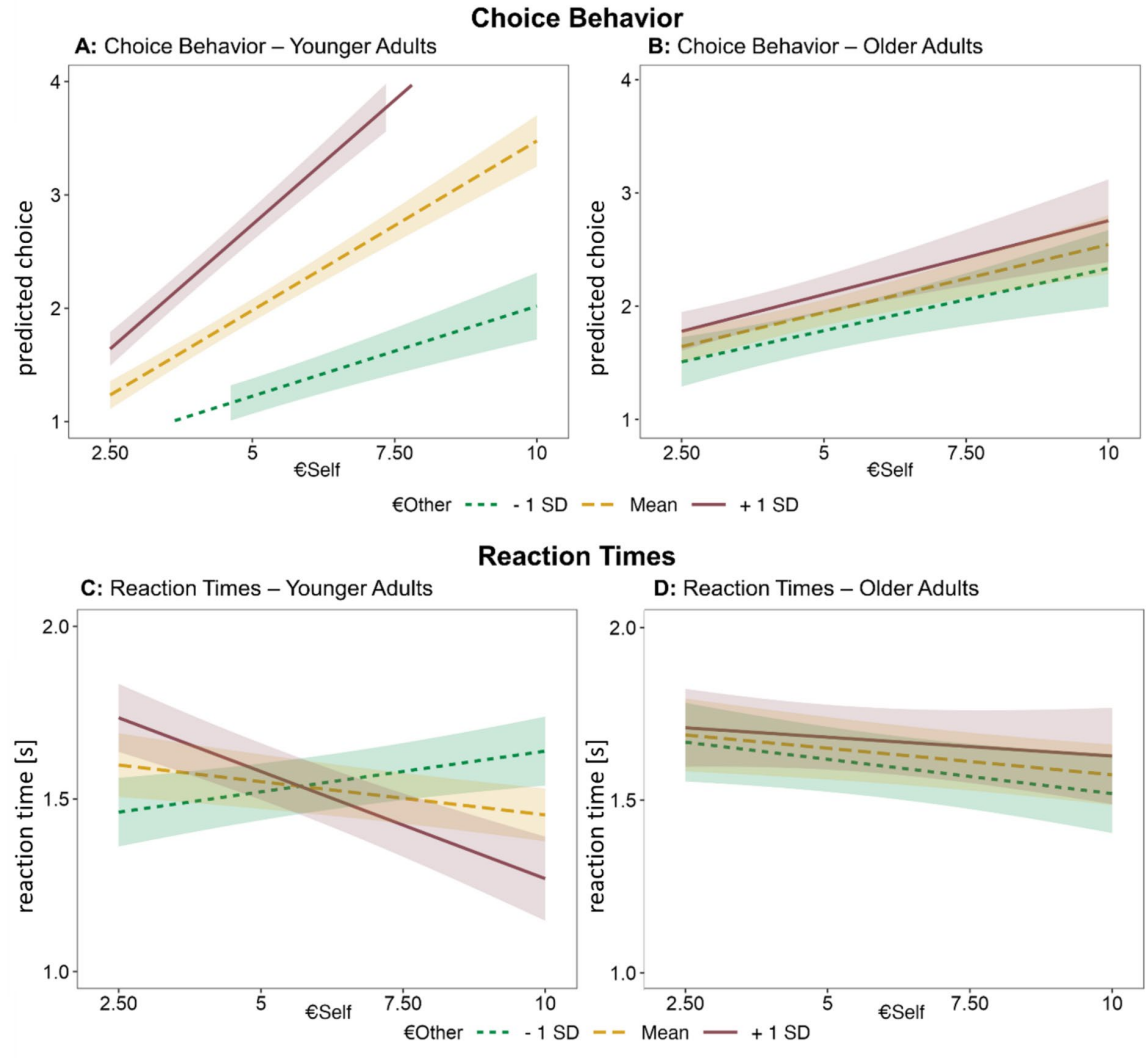
Adult age group differences in choice behavior and RTs with respect to the interaction of €self × €other. Note. We identified significant 3-way interactions of age group × €self × €other (both for choices and RTs). The shaded areas around the lines represent the 95% confidence intervals. Note that there was no condition where both values (i.e., €self and €other) fell below 5€ (**A** and **B**) Predicted frequency of accepting the alternative offer. Here, the y-axis corresponds to the four choice options reflecting the extent to which the alternative offer is preferred (1 = strong no, 2 = weak no, 3 = weak yes, 4 = strong yes). Younger adults’ choices were modulated by an interaction of €self × €other. Higher values increased the probability of choosing the proposed offer, i.e., choosing either 3 or 4. Older adults’ tendency to accept an offer as a function of own benefits was not significantly modified by payoffs for the other: The probability of choosing the proposed offer increased with higher values of €self (regardless of €other). (**C**) Younger adults’ RTs were modulated by an interaction of €self × €other. They showed faster RTs when both values (€self and €other) were either high or low, and slower RTs when values diverged (i.e., one was high and the other low). (**D**) Older adults’ RTs were only modulated by €self, irrespective of €other. Faster RTs were observed for higher values of €self (regardless of €other).

We were particularly interested in whether adult age impacted (pro)social choice behavior with respect to the degree to which outcomes for oneself (€self) and others (€other) guide behaviors. We calculated a mixed effect regression model with trial-by-trial choices as dependent variable, and the main effects and interactions of the following predictor variables: age group, payoffs to oneself (€self), and payoffs to the partner (€other). We observed the expected main effects of age group, €self, and €other (all *p*s < 0.002, see Table 3) (compare^18^), as well as significant age × €self, and age × €other iteractions (all *p*s < 0.001) on a decision to accept an offer. Critically, we additionally found a significant 3-way interaction of age group × €self × €other (*F*(109.02) = 30.80, *p* < 0.01). The latter indicates age group differences with respect to the integration of €self and €other (see Fig. 2A and B).

**Table 3:** *Mixed effect model of choice behavior predicted by age group x €self €other interaction.*

	DF	F-value	*p*-value	Beta	d
Intercept				2.32	
age group	109.00	10.55	0.002**	-0.16	0.18
€self	109.00	214.14	< 0.001***	0.21	0.24
€other	109.02	129.65	< 0.001***	0.16	0.19
age group x €self	109.00	39.05	< 0.001***	-0.09	0.10
age group x €other	109.02	63.05	< 0.001***	-0.11	0.13
€self x €other	109.02	41.11	< 0.001***	0.02	0.02
age group x €self x €other	109.02	30.80	< 0.001***	0.02	0.02

As a post-hoc test, the data was split into two models, separately for each age group, and including the main effects, as well as the 2-way interaction of €self × €other. We found significant main effects of €self and €other in both younger as well as older adults (all *p*s < 0.02, see Table S2 for full results). Interestingly, however, while younger adults’ choices were modulated by an interaction of €self × €other (*F*(61.99) = 62.71, *p* < 0.001), this was not observed in older adults’ choice behavior (interaction term €self × €other : *F*(47.10) = 0.57, *p* = 0.46). As Fig. 2A illustrates, in younger adults, the tendency to choose the proposed offer as a function of €self was modulated by €other: Indeed, low payoffs for the other person decreased the tendency to accept an offer with increasing €self in younger adults compared to high payoffs for the other (simple slope €other – 1SD: *β* = 0.16, 95% interval = [0.11, 0.21]; simple slope €other + 1SD: *β* = 0.44, 95% interval = [0.39, 0.49], contrast €other − 1SD vs. + 1SD: estimate = -0.28, *p* < 0.001, see Fig. 2A). In older adults, the probability of accepting the proposed offer increased with higher values of €self, irrespective of €other (simple slope €other – 1SD: *β* = 0.11, 95% interval = [0.06, 0.16]; simple slope €other + 1SD: *β* = 0.13, 95% interval = [0.07, 0.19], contrast €other − 1SD vs. + 1SD: estimate = -0.02, *p* = 0.73, see Fig. 2B). That is, whilst there was evidence for an additive effect of €self and €other (i.e., significant main effects of €self and €other), there was no significant evidence for an integration of both values (in the sense of a multiplicative effect) in older adults.

### Reaction times

Next, we examined age-related differences in the tradeoff between self- and other-regard (i.e., interaction €self × €other) captured in RTs in younger versus older adults. Again, we calculated a mixed effect model, consisting of trial-by-trial RTs as dependent variable, and the same predictors of age group, value for self (€self), and value for other (€other), including their main effects and interactions. Strikingly, the RT results mirrored the pattern we observed for choice behavior: the 3-way interaction of age group × €self × €other was significant (*F*(108.94) = 18.30, *p* < 0.001, see Table S3 and Fig. 2C and D). Again, younger adults’ RTs were modulated by a significant 2-way interaction of €self × €other (*F*(61.96) = 27.37, *p* < 0.001, see Table S4), while this was not apparent in older adults‘ RTs (*F*(46.95) = 0.44, *p* = 0.51, see Table S4). The significant 2-way interaction of €self × €other in younger adults illustrates that they reacted quickly when values for €self and €other were either both high or both low, and younger adults reacted slowest when there was a conflict between €self and €other, i.e., when one of them was high, and the other was low, or vice versa (simple slope €other – 1SD: *β* = 0.02, 95% interval = [0.01, 0.04]; simple slope €other + 1SD: *β* = -0.06, 95% interval = [-0.08, -0.04], contrast €other − 1SD vs. + 1SD: estimate = 0.09, *p* < 0.001, see Fig. 2C). Older adults on the other hand reacted faster the higher €self was in a trial’s offer, and this was not significantly influenced by €other (simple slope €other – 1SD: *β* = -0.02, 95% interval = [-0.04, -0.002]; simple slope €other + 1SD: *β* = -0.01, 95% interval = [-0.03, -0.01], contrast €other − 1SD vs. + 1SD: estimate = -0.01, *p* = 0.79, see Fig. 2D).

### Moderation effect of inhibitory control on choice behavior

In the main manuscript, moderation effects in terms of significant 3-way interactions for choice behavior (i.e., moderator × €self × €other) are reported. Results of other moderator analyses (including social, and general cognitive moderators) are provided in the supplemental results (see pp.6–13). See Figures S4 and S5 for histrograms of the distribution of the different moderator variables.

**Fig. 3:**
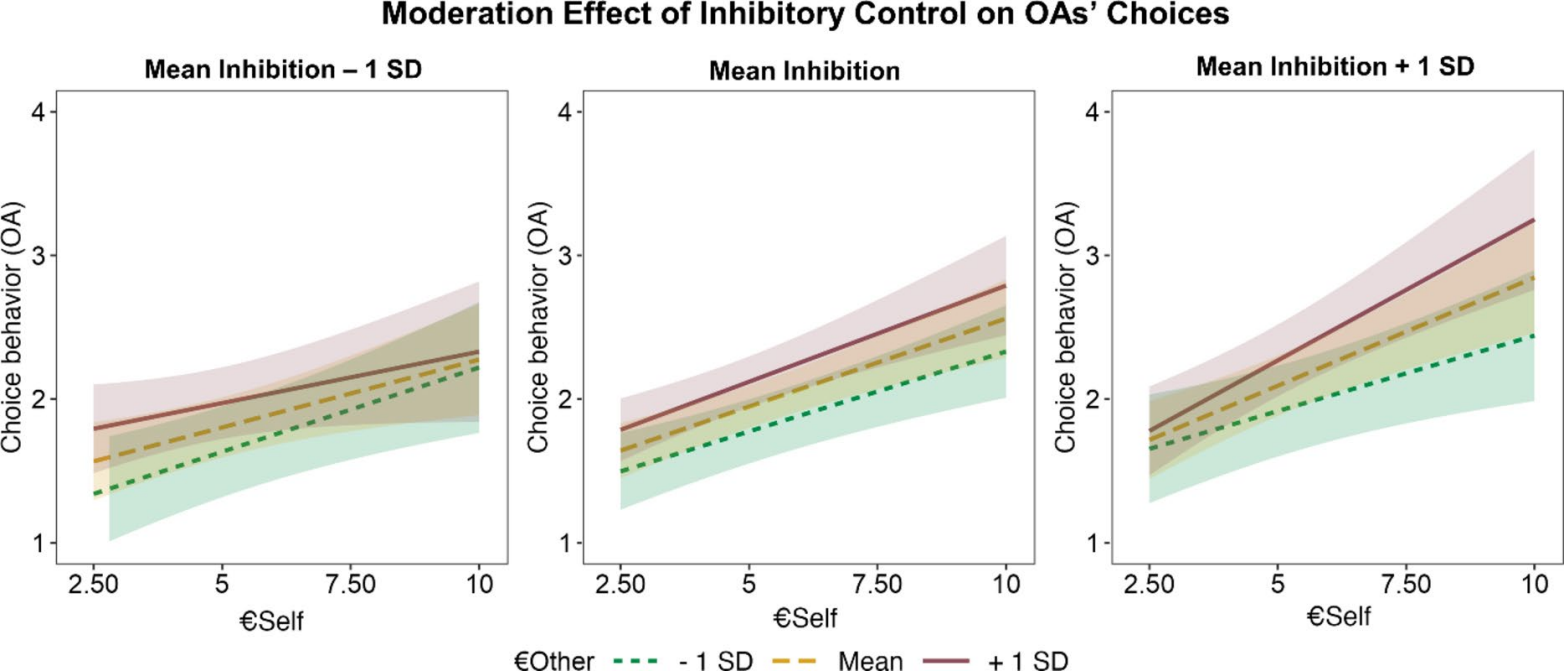
Significant moderation effect of inhibitory control on older adults’ choices behavior (mean inhibitory control – 1 standard deviation vs. mean inhibitory control vs. mean inhibitory control + 1 standard deviation). Here, the y-axis corresponds to the four choice options reflecting the extent to which the alternative offer is preferred (1 = strong no, 2 = weak no, 3 = weak yes, 4 = strong yes). Note. Significant interaction of €self x €other x inhibition ability. Note that we split up the sample in low vs. medium vs. high inhibitory control for illustration purposes only. Inhibition ability entered the model as a continuous predictor. In older adults with high inhibition abilities (right panel, mean inhibitory control + 1 SD), choices to accept the proposed offer were impacted by a multiplicative effect of both €self and €other. In older adults with medium inhibition abilities (middle panel, mean inhibitory control), choices to accept the proposed offer were impacted by values for €self and €other, but not by the interaction of €self x €other. Older adults with low inhibition abilities (left panel) were only influenced by the values for self (€self). The shaded areas around the lines represent the 95% confidence intervals. SD = standard deviation, OA = older adults.

In younger adults, we did not observe significant main or interaction effects involving inhibitory control (all *p*s > 0.25, see Table S5 for full results). Interestingly, we found a significant moderation effect of inhibitory control on older adults’ choices (i.e., significant 3-way interaction of inhibition × €self × €other, *F*(42.04) = 6.27, *p* = 0.02, see Fig. 3 and Table S5 for visualization and full results). As Fig. 3 suggests, older adults with higher inhibitory control were better able to integrate payoffs for themselves and others. That is, in the older adults subgroup with high inhibitory capacities (see Fig. 3, right panel, mean inhibitory control + 1 SD), slopes for €self differed as a function of €other (*β* = 0.01, *SE* = 0.01, 95% confidence interval [0.002, 0.022]). In other words, as can be seen in Fig. 3 (right panel), for the subgroup with high inhibitory control abilities, the response pattern looked more similar to younger adults’ response pattern compared to the subgroup with lo inhibitory control abilities. Older adults with medium inhibitory control abilities (see Fig. 3, middle panel, mean inhibitory control) showed a positive effect of €self (*β* = 0.12, *SE* = 0.02, 95% confidence interval [0.08, 0.17]) and €other (*β* = 0.05, *SE* = 0.02, 95% confidence interval [0.01, 0.09]), but no interaction of €self × €other (*β* = 0.003, *SE* = 0.004, 95% confidence interval [-0.004, 0.011]). Interestingly, in older adults with low inhibitory capacities (see Fig. 3, left panel, mean inhibitory control – 1SD), there was a positive effect of €self (*β* = 0.10, *SE* = 0.03, 95% confidence interval [0.03, 0.16]), but not €other (*β* = 0.04, *SE* = 0.03, 95% confidence interval [-0.02, 0.09]), and the positive effect of €self was not significantly modulated by €other (*β* = -0.01, *SE* = 0.01, 95% confidence interval [-0.02, 0.004]).

### Hierarchical drift-diffusion model

We conducted hierarchical drift-diffusion modeling analyses to determine which components of the social decision-making process were differentially influenced by condition, and the trade-off between self-regard (€self) and other-regard (€other) in the two age groups.

We estimated the three main DDM parameters for every participant (*v*-, *z*-, and *a*-parameter) within the same model, but separately for younger and older adults. Across 12 models per age group, the winning model with the lowest DIC value was the most complex model that was subsequently chosen to test our hypotheses (see Table 1). This model allowed the *a*- (boundary separation) and *z*-parameter (initial bias) to vary by condition (other-serving vs. self-serving vs. rational). The *v*-parameter (drift rate) was assumed to be modulated by payoffs for participants themselves (€self), for the partner (€other), and their interaction (€self × €other). We further modeled the non-decision time *ndt* as a fixed parameter across conditions (YA: *M* = 0.98, *SD* = 0.21; OA: *M* = 0.92, *SD* = 0.29). By comparing our observed data with 500 simulated datasets, we could illustrate that the winning model fits the data well, both in younger and older adults (see Tables S12-S13).

Here, we were particularly interested in whether age group differences in the integration of values for €self and €other can be explained by age group differences in drift rate (*v*-parameter). The winning DDM further indicated a modulation of the *z*-parameter and the *a*-parameter by the different conditions. These results are reported in the Supplement. Moreover, linear models that were conducted to examine whether the pre-defined moderators (inhibitory control, cognitive functioning, empathy, compassion, and ToM) predicted the mean group posteriors of the v-, z-, and a-parameter are also included in the supplementary results (pp. 19, Tables S9 – S11). In brief, we found significant associations of the a-parameter and ToM-abilities in younger adults, and correlations of boundary separation with cognitive abilities (fluid and verbal) in older adults (for more details see supplementary results section).

In younger adults, we observed meaningful main effects of €self and €other on drift rate (*v*-parameter). The drift rate increased in proportion to the degree of €self (probability(v_*YA−€self*_ > 0) = 1.0) and €other (probability(v_*YA−€other*_ > 0) = 0.96) (see Fig. 4A). Matching main effects of €self and €other were also present in older adults (probability(v_*OA−€self*_ > 0) = 1.0; probability(v_*OA−€other*_ > 0) = 1.0; see Fig. 4A). Notably, comparisons of main effects between younger and older adults did not reveal differences across age groups (probability(v_*YA−€self*_ > v_*OA−€self*_) = 0.91; probability(v_*YA−€other*_ > v_*OA−€other*_) = 0.75).

Next, we investigated the influence of the integration of values for €self and €other (i.e., the interaction term €self × €other) on drift rates in younger and older adults. Younger adults’ drift rates were positively impacted by the interaction of €self × €other (probability(v_*YA−€selfx€other*_ > 0) = 1.0; see Fig. 4B). Interestingly, no such interaction effect on older adults’ drift rates was observed (probability(v_*OA−€selfx€other*_ > 0) = 0.51, see Fig. 4B). Direct comparisons across age groups found that the influence of the interaction of €self × €other on drift rates was meaningfully more pronounced in younger compared to older adults (probability(v _YA−€selfx€other_ > v _OA−€selfx€other_) = 1.0; see Fig. 4B).

Taken together, consistent with previous findings^18,52,113^, values of €self and €other were linked to drift rates *v* (the speed of information accumulation) and, thus, the efficiency of the social decision-making process. This was true for both age groups. However, drift rates in younger adults – but not older adults – were additionally associated with the interaction of both payoffs (€self × €other), indicating differences in the complex trade-off and integration of information of benefits for €self and €other across age groups.

Testing the relationship between individuals’ modulation of the v-parameter by an interaction of €self and €other with individual Fehr-Schmidt preferences, revealed no meaningful association between these two characteristics of the social decision-making process (rho = 0.16, S = 99106, *p* = 0.14, BF = 0.37).

In addition to the drift-rate parameter, which was the principal parameter of interest, our model comparison showed that a model in which the initial bias and the boundary separation parameters were modulated by the information about which condition participants were facing. In summary, younger adults demonstrated a larger initial bias towards rejecting self-serving offers than rejecting other-serving offers (probability(*β-z*_YA−self−serving_ < 0) = 0.96), whereas older adults showed a larger initial bias towards accepting self-serving offers than accepting other-serving offers (probability(*β*-z_OA−self−serving_ > 0) = 1.0). With regard to the boundary separation, results showed no meaningful differences between the age groups, but both age groups needed to accumulate less evidence to reach a decision in the self-serving as compared to the other-serving condition(probability(*β-a*_YA−self−serving_ < 0) = 1.0; probability(*β-a*_OA−self−serving_ < 0) = 1.0). The detailed results of these parameters’ posterior distributions and corresponding analyses are reported in the Supplementary Material (pages 20–25).

### Bayesian posterior densities of drift rates (v-parameter)

**Fig. 4:**
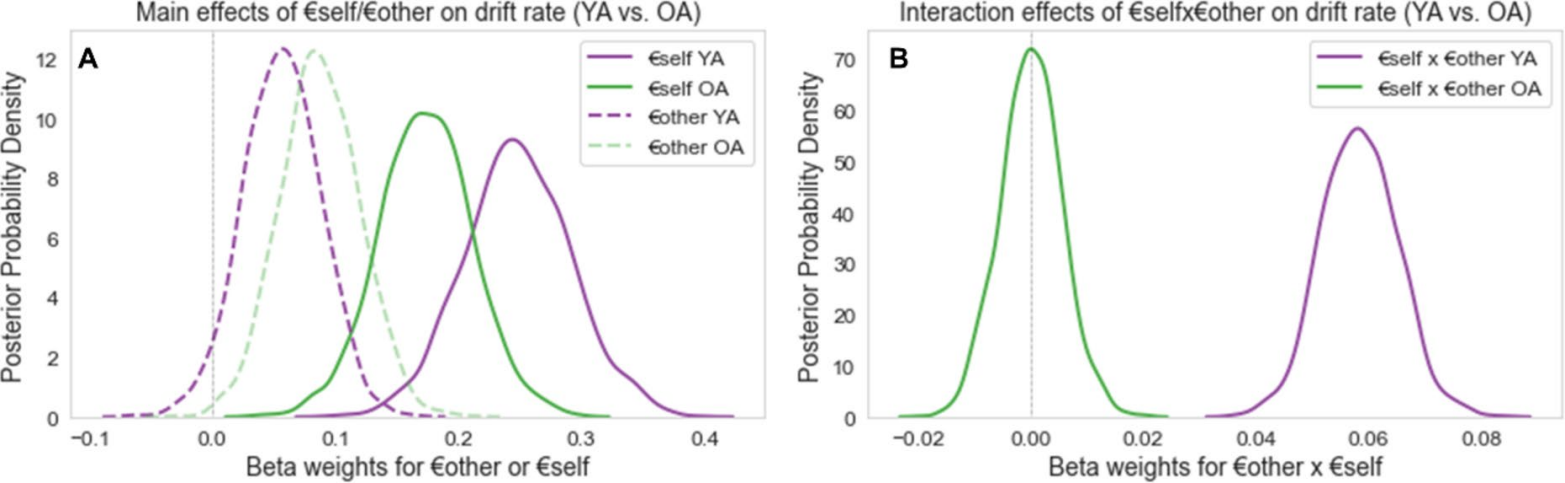
Differential effects of €self, €other and their interaction on drift rate in younger and older adults. Note. Bayesian posterior densities of drift rates (*v*-parameter) estimated from hierarchical regression drift-diffusion models and how they varied as a function of payoff values €self and €other. For an effect to be meaningful, 95% of the distribution has to be on the left or right of zero (dotted vertical grey line). (A) Main effects of €self and €other on the drift rate, separately for younger (YA; purple) and older adults (OA; green). All main effects indicated a positive impact of €self and €other on drift rates in YA and OA. (B) €self × €other interaction effect on the drift rates in YA and OA. Only younger adults demonstrated a positive interaction effect of €self × €other on drift rates.

## Discussion

Here, we adopted a value-based decision-making framework and applied hierarchical drift-diffusion modeling to investigate age group differences in decision-making for self and others. Other than previously suggested, older adults did not display higher prosociality per se in our study. Harvesting our design, we however found differences in how younger and older adults solve the cost-benefit tradeoff of (pro)social decision-making. We found that younger, but not older adults, integrated values for self and others in a multiplicative fashion: the tendency to accept an offer based on their own payoff was modulated by the others’ payoff only in younger adults. Interestingly, older adults with better inhibitory control abilities demonstrated improved integration of the different values for €self and €other, showing a behavioral pattern that qualitatively resembled the behavior of younger adults. These findings provide novel mechanistic insights into *how* age might change the social decision process and unravel inhibitory control as an important moderator of age-related differences in prosocial decision-making. Computational modelling showed that the integrated information of values for self and others (€self, €other) positively influenced the efficiency of younger adults’ decision-making process. Contrary, in older adults, payoffs for self and others affected decision efficiency in an additive, but not an interacting, fashion.

Previous meta-analytic evidence suggests a small age-related increase in self-reported and behavioral prosociality across adulthood, but also substantial heterogeneity in findings^9^. In the current study, we did not observe increased prosociality per se in older versus younger adults. Even though the prevailing view in the literature is that older people are more prosocial than young people, the present results are in line with a recently published study using a similar behavioral paradigm^126^, that observed less aversion towards advantageous inequity in older compared to younger adults. Moreover, additional recent studies demonstrated a negative association between age and prosociality, i.e. decreased prosocial behavior in older age^127,128^, painting a more mixed picture of prosociality in older age than has traditionally been suggested.

We used drift-diffusion modeling to gain further insight into the underlying processes of these age group differences in social value integration. Mirroring choice data and RT results, modeling indicated that for younger adults, an interaction of values for self and others was associated with a more efficient decision-making process, characterized by a more rapid accumulation of evidence towards accepting an offer. Previously, a formal model of evidence accumulation in prosocial decision-making^18^, based on data in younger adults, tested an additive effect of monetary gain for the self and the other on the decision-making process. That is, the information about how much the participant receives herself and how much the other person receives is considered independently from each other in order to come to a conclusion. In this additive approach, information about €self and €other are thus not integrated. Our study extends this model by showing that younger adults show a multiplicative effect while older adults’ decision-making efficiency was influenced by self or other monetary gain in an additive fashion, i.e., separately considering outcome information for the self and the other. Thus, in the present study, younger adults do integrate the information about €self and €other such that the weight of €self depends on the value of €other and vice versa. This means, for example, that a low gain of €other can devalue a high potential gain €self for that decision option. We observe this multiplicative effect in younger adults’ choice and RT data as well as in the speed of evidence accumulation.

Work outside of social contexts has previously reported age differences between younger and older adults in certain tasks that involved combining information from different sources, (e.g., perception^56,129^), or making decisions based on cost-benefit analyses^11,19,21^. In these studies, older individuals were typically shown to be impaired in a capacity to effectively integrate all relevant information^23,130^. It is concluded that in order to reduce the cognitive demands of a specific task^26,27^, older individuals adapted their decision-making behavior by analyzing and integrating less information^28–30^ and showing less efficient decision-making when information needs to be integrated^56^ or when more complex memory processes are required^57,58^. According to the resource-rational approach^21^, the employment of simpler and less demanding decision approaches (such as reduced information integration), present in children and older individuals, can be seen as a deliberative and effective decision-making strategy optimizing the use of available mental resources. Thus, the present results extend these previous findings to the realm of social decision-making by demonstrating relatively less consideration of integrated information in older as compared to younger adults. More generally, our results argue in favour of conceptualizing prosocial behavior as a cost-benefit computation. For aging studies, they suggest experimental designs akin to ours in which value for self and other are parametrically manipulated and traded off against each other. Given that older adults showed a general tendency to favour offers (both self-serving and other-serving offers), we speculate that other tasks (e.g., one-shot tasks) could yield misleading results, depending on response framing (e.g., if saying “yes” or “accept”  is associated with helping or giving in a study^131^).

The present study also showed that older individuals with better inhibitory control abilities demonstrated a greater capacity to effectively integrate values for self and others. Inhibitory control enables flexible decision-making in response to changing task demands^132,133^ and studies consistently indicated a decline in inhibitory control abilities with older age^77,79,134,135^. Most of the existing research linking inhibitory control and prosocial behavior focused on the period of childhood^136–138^, indicating that children with better inhibitory control tended to exhibit higher levels of prosocial behavior. There is a lively debate whether prosociality in adults is intuitive^83,139,140^, or whether before acting prosocially, individuals must overcome prepotent, selfish impulses^137,138^. Additional lifespan research is thus needed to gain more insights into the relationship between inhibitory control, prosocial decision-making, and its underlying mechanisms.

Moreover, contrary to what we expected based on the literature, drift diffusion modeling demonstrated that older adults showed an initial bias towards rejecting other-serving offers (i.e., offers that benefitted partners at a cost to themselves) and their initital bias suggested a tendency to accept self-serving offers (i.e., offers that benefitted themselves at the partner’s cost). Younger adults,however, showed a different pattern of initital bias.They did not express a clear bias towards accepting or rejecting other-serving offers and instead displayed a bias towards rejecting self-serving offers.

Hence, the current results are inconsistent with some typical assumptions about prosociality in younger and older age groups. Firstly, they challenge the conception of younger individuals, particularly emerging adults, as inherently selfish^141^, as they did not exhibit a bias favoring self-serving offers. Secondly, they do not align with a view of older individuals as inherently prosocial^142^, as they did neither show a bias towards other-serving offers in older adults, nor did our choice data indicate more generous choice behavior in older adults during the dictator game in general. Interestingly, there is evidence arguing that an initial bias reflects personal predispositions^50^, but can also be distorted by social information (e.g., about the interaction partner^143,144^), something we cannot disentangle with the current design.

A limitation of our study is that, when using the drift diffusion model, we used a two decision options approach to model data with four decision options by binarizing the data. This was done to reflect the decision-outcome relationship, where the implementation of the participant’s decision (on a 4-point-scale) was binary. This approach was based on other studies using the same task^18,51^. In future studies other models such as the Linear ballistic model^145^ or models taking into account the confidence with which a decision was made^146–148^ could shed more light on additional aspects of the decisions process without focussing on the integration of the different point-values or general prosociality. Within the current study, we do not account for a potential influence of these processes. Further, it may be that the older adults included in this study are financially better off than the young adults in this study, who are often students with limited income. Indeed, differences in socio-economic status have been shown to be associated with behaviour in a dictator game with monetary incentives^149^, and a recent meta-analysis has shown that lifespan age differences in pro-sociality were attenuated when participants were not playing for real money in neuro-economic games^150^. However, note that these findings would lead to a prediction that differs from the findings observed in this study, namely that older adults are more generous because they are wealthier. Still, in future studies on adult age differences in prosociality, it would be valuable to focus on behaviours in which costs and benefits do not differ as a function of age group or can be individually adapted^151^. A further limitation is that in this study we only tested prosocial behaviour towards a member of the same age group. Indeed, previous studies have found that the age of the social partner influences social cognition in older and younger adults in social decision-making tasks^e.g., 143^. In future studies, it would be a valuable addition to manipulate the age of the recipient in the dictator game to test for interaction effects of own age and the other’s age on the integration of values for self and other.

Further, we compared only younger and older adults, leaving out the socially important phase of midlife. There are reasons to hypothesize that social aspects, such as prosociality and empathy, undergo changes during midlife^9^. The period of midlife may be a particularly prosocial and empathetic phase as suggested by theoretical approaches, research on personality traits, as well as typical developmental tasks including caregiving^9,75,152–158^. Thus, including this interesting and complex period of life in future studies would further contribute to the understanding of the adult development of a relationship between socio-emotional factors and prosocial behavior.

## Conclusion

In summary, by adopting a value-based decision framework and utilizing drift diffusion modeling, we uncovered age-related differences in how older and younger individuals integrate values for self and others to reach a social decision. Collectively, this study provides valuable insights into the behavioral and computational mechanisms underlying age differences in social decision-making processes. It emphasizes social decision-making as a cost-benefit analyses which is solved differently by younger and older adults. In conclusion, these findings underscore the significance of exploring the underlying (computational) mechanisms of prosocial behavior and the broader social decision-making process to develop a comprehensive understanding of how this construct evolves throughout adulthood.

## Electronic supplementary material

Below is the link to the electronic supplementary material.

Supplementary Material 1

## Data Availability

All data and materials are publicly available at: https://osf.io/zu4p3/.
