# Supplementary material for: Adult age differences in the integration of values for self and other
Source: Sci Rep. 2025 Apr 14;15:12776. doi: 10.1038/s41598-025-96656-6 (PMC11997085; doi:10.1038/s41598-025-96656-6)
Supplement: Supplementary file 1 — Supplementary Material 1 [file 41598_2025_96656_MOESM1_ESM.docx]

**Supplemental Materials**

**Accompanying the manuscript “Adult Age Differences in the Integration of Values for Self and Others”**

# Methods

## Recruitment and exclusion criteria

Participants were recruited via different databases of the TU Dresden and flyers and newspaper announcements in Dresden (Germany). Additionally, older adults were recruited from local sports, language, and university courses and choirs. A-priori inclusion criteria were defined as follows: right-handed, fMRI suitable (e.g., no magnetic implants), fluency in German with normal or corrected-to-normal vision, no hearing deficits, no color-blindness, no self-reported psychological or neurological diseases presently or within the past 12 months, and no history of drug abuse. Additionally, participants were not allowed to consume more than five cups of coffee per day, more than five cigarettes per day, more than 12g (women)/24g (men) pure alcohol per day, or illegal drugs more than twice per month. Older adults underwent an additional screening for mild cognitive impairments with the German version of the Montreal Cognitive Assessment (MOCA; Nasreddine et al., 2005). Only older adults with a score of at least 26 points (out of 30) were included (see also Stietz et al., 2021). During or after the third session of the experiment, a total of 15 participants were excluded based on the following reasons: inability to undergo the scanning procedure (*n* = 8), neuromorphological abnormalities detected through MRI (*n* = 4), missing rate in our experimental task higher than 10% (*n* = 3). The final sample included 63 younger adults (31 females, age range = 18-30, *M* = 24.42 years, *SD* = 3.30) and 48 older adults (24 females, age range = 65-78, *M* = 69.71 years, *SD* = 3.84)

**Table S1.** *Descriptive and inferential statistics with respect to sample characteristics, inhibition scores, cognitive functioning, and the EmpaTom measures.*

| Sample Characteristics | *n* (YA / OA)^1^ | Frequency (YA / OA)^2^ | Test statistic |
| --- | --- | --- | --- |
| Relationship status (partner\|single) | 62/44 | 13\|31 / 27\|35 | *χ*^2^ = 1.59, *p* = 0.21 |
| Residence (alone\|with others) | 61/48 | 14\|47 / 14\|34 | χ^2^ = 2.10, *p* = 0.35 |
| Sample Characteristics | *n* (YA/OA) | M (*SD*) (YA/OA) | Test statistic |
| Years of education | 62/41 | 16.71 (2.28) / 16.84 (3.15) | *t* = 0.39, *p* = 0.69 |
| Inhibition |  |  |  |
| Go-Nogo (ms) | 63/47 | 388 (88) / 461 (105) | *t* = 4.06, *p* < 0.01 |
| Stop Signal (ms)* | 60/46 | 184 (55) / 210 (60) | *t* = -2.27, *p* = 0.03 |
| Stroop (ms) | 63/45 | 71 (49) / 128 (93) | *t* = 3.18, *p* < 0.01 |
| Inhibition composite score (ms) | 60/44 | 0.27 (0.63) / -0.35 (0.70) | *t* = 4.92, *p* < 0.01 |
| Cognitive Functioning |  |  |  |
| IDP accuracy | 63/47 | 0.96 (0.05) / 0.96 (0.05) | *t* = 0.27, *p* = 0.79 |
| IDP RT (ms) | 63/47 | 2113 (333) / 3109 (457) | *t* = 12.82 *p* < 0.01 |
| SAW accuracy | 63/47 | 0.71 (0.07) / 0.81 (0.09) | *t* = 7.42, *p* < 0.01 |
| SAW RT (ms) | 63/47 | 3642 (1022) / 3779 (745) | *t* = 1.29, *p* = 0.20 |
| TMT A (sec) | 63/47 | 22.41 (7.09) / 40.82 (17.32) | *t* = 9.33, *p* < 0.01 |
| TMT B (sec) | 63/47 | 44.81 (13.85) / 82.13 (27.84) | *t* = 9.75, *p* < 0.01 |
| DSb | 63/47 | 7.56 (2.26) / 6.28 (1.41) | *t* = 2.59, *p* = 0.01 |
| Composite of fluid abilities | 63/47 | 0.42 (0.41) / -0.57 (0.50) | *t* = 10.26, *p* < 0.01 |
| Composite of verbal abilities | 63/47 | -0.20 (0.66) / 0.26 (0.62) | *t* = 4.52, *p* < 0.01 |
| Social emotions and understanding (EmpaToM) | | | |
| Empathy | 63/48 | -0.61 (0.41) / -0.66 (0.59) | *t* = 0.98*, p* = 0.33 |
| Compassion | 63/48 | 3.19 (0.93) / 3.78 (1.25) | *t* = 5.88*, p <* 0.01 |
| Theory of Mind | 63/48 | 0.71 (0.18) / 0.50 (0.20) | *t* = 7.07*, p < 0.01* |

*Note.* YA = Younger adults, OA = Older adults, RT = Reaction time, IDP = Identical Pictures test, SAW = Spot A Word test, TMT A = Trail Making test A, TMT B = Trail Making test B, Dsb = Digit Span backward test. Test statistics represent results from a non-parametric robust yuen test (see Mair & Wilcox, 2019) due to violations with respect to the assumption of normality and/or homoscedasticity.

^1^These sample sizes indicate the number of YA and OA who reported their relationship status, residence, and years of education, and were consequently included in the statistical analyses (see test statistics).

^2^The frequencies correspond to the relationship status (i.e., partner vs. single) and the residence categories (i.e., alone vs. with others). The pipe distinguishes the different response categories, while the slash distinguishes between YA and OA.

*Results from an independent t-test are reported as the assumptions of normality and homoscedasticity are fulfilled.

# Results

**Mixed effect model of choice behavior and RTs**

**Table S2.** *Post-hoc mixed effect models of choice behavior predicted by €self x €other interaction separately in younger and older adults.*

|  | Younger Adults | | | |  | Older Adults | | | |  |
| --- | --- | --- | --- | --- | --- | --- | --- | --- | --- | --- |
|  | DF | F-value | p-value | Beta | d | DF | F-value | p-value | Beta | d |
| Intercept |  |  |  | 2.48 |  |  |  |  | 2.16 |  |
| €self | 61.99 | 276.90 | < 0.001*** | 0.30 | .42 | 47.00 | 27.70 | < 0.001*** | 0.12 | .12 |
| €other | 62.00 | 191.09 | < 0.001*** | 0.28 | .39 | 47.03 | 6.32 | 0.02* | 0.05 | .05 |
| €self x €other | 61.99 | 62.71 | < 0.001*** | 0.04 | .05 | 47.10 | 0.57 | 0.46 | 0.003 | .003 |

***Table S3.*** *Mixed effect model of RTs predicted by age group x €self x €other interaction.*

|  | DF | F-value | p-value | Beta | d |
| --- | --- | --- | --- | --- | --- |
| Intercept |  |  |  | 1.57 |  |
| age group | 108.98 | 3.24 | 0.08 | 0.05 | 0.16 |
| €self | 108.95 | 18.08 | < 0.001*** | -0.02 | 0.05 |
| €other | 108.72 | 0.01 | 0.94 | -0.0004 | 0.001 |
| Trial | 19447.14 | 2731.61 | < 0.001*** | -0.003 | 0.01 |
| age group x €self | 108.95 | 0.23 | 0.64 | 0.002 | 0.006 |
| age group x €other | 108.73 | 7.88 | 0.01** | 0.02 | 0.04 |
| €self x €other | 108.94 | 12.03 | < 0.001*** | -0.005 | 0.02 |
| age group x €self x €other | 108.94 | 18.30 | < 0.001*** | 0.007 | 0.02 |

**Table S4.** *Post-hoc mixed effect model RTs predicted by €self x €other interaction separately in younger and older adults*

|  |  | Younger Adults | | | | | | Older Adults | | | | |  | |
| --- | --- | --- | --- | --- | --- | --- | --- | --- | --- | --- | --- | --- | --- | --- |
|  | DF | | F-value | p-value | Beta | d | DF | | F-value | p-value | Beta | d | |  |
| Intercept |  | |  |  | 1.52 |  |  | |  |  | 1.62 |  | |  |
| €self | 61.99 | | 11.53 | 0.001** | -0.02 | 0.07 | 46.92 | | 7.48 | 0.009** | -0.02 | 0.04 | |  |
| €other | 61.82 | | 3.64 | 0.06 | -0.01 | 0.04 | 47.05 | | 5.73 | 0.02* | 0.01 | 0.03 | |  |
| Trial | 11037.27 | | 1749.98 | < 0.001*** | -0.004 | 0.01 | 8417.95 | | 1003.55 | < 0.001*** | -0.003 | 0.01 | |  |
| €self x €other | 61.96 | | 27.37 | < 0.001*** | -0.01 | 0.04 | 46.95 | | 0.44 | 0.51 | 0.001 | 0.003 | |  |

## Moderation Regression Analyses on Choice Behavior and Reaction Times

**Moderation effect of inhibitory control on reaction times**

No significant main or interaction effects involving the moderator of inhibitory control on RTs were observed in younger or older adults (all ps > 0.07, see Table S6)

### **Moderation effect of cognitive functioning on choice behavior**

**Composite of fluid abilities.**

We did not observe significant main or interaction effects, including the composite score of fluid abilities, in neither younger adults’ (all adj. *p*s > 0.39, see Table S5), nor older adults’ (all adj. *p*s > 0.07, see Table S5) choice behavior.

**Composite of verbal abilities**. For both, younger (all adj. ps > 0.07, see Table S5) and older adults (all adj. ps > 0.08, see Table S1) we did not find significant main or interaction effects for the composite score of verbal abilities.

### **Moderation effect of cognitive functioning on reaction times**

**Composite of fluid abilities.**

We did not find significant main or interaction effects, including the composite score of fluid abilities in younger adults (all adj. *p*s > 0.14, see Table S6) and older adults (all adj. ps > 0.10, see Table S6).

**Composite of verbal abilities.** No main or interaction effects involving the Spot A Word test (SAW) as a measure of verbal abilities were found for older adults’ RTs (all adj. *p*s > 0.42, see Table S6). Interestingly, for young adults’ RTs, a significant moderation effect in terms of a significant 3-way interaction of SAW × €self × €other was observed (*F*(60.95) = 8.29, adj. *p* = 0.01, see Figure S1 and Table S6). As indicated in Figure S1, both groups showed the same pattern, meaning quicker RTs when values for self and other were either both high or both low, and slower RTs when there was a conflict, i.e., only one of them was high and the other low (or vice versa). However, the RT differences were particularly pronounced in younger adults with lower verbal abilities, who showed a slowing of RTs with higher €other (simple slope SAW - 1SD & €other – 1SD: *β* = 0.05, 95% interval = [0.03, 0.07]; simple slope SAW -1SD & €other + 1SD: *β* = -0.08, 95% interval = [-0.11, -0.05], see Figure S1, left panel), compared to younger adults with higher verbal abilities (simple slope SAW + 1SD & €other – 1SD: *β* = -0.001, 95% interval = [-0.02, 0.02]; simple slope SAW +1SD & €other + 1SD: *β* = -0.04, 95% interval = [-0.07, -0.01], see Figure S1, right panel). No further main or interaction effects involving SAW were observed in younger adults (all adj. *p*s > 0.14, see Table S6).

**Figure S1**

*Significant moderation effect of verbal abilities (*Spot A Word test; *SAW) in younger adults’ RTs on the integration of €self × €other*

*Note.* In younger adults (YA) with lower verbal abilities (left panel), the interaction of €self × €other was more pronounced compared (right panel) to YA with higher verbal abilities. The shaded areas around the lines represent the 95% confidence intervals.

### **Moderation effect of empathy, compassion, and theory of mind on choice behavior**

**Empathy & compassion.**

No statistically significant main or interaction effects, including empathy or compassion, were observed in younger adults’ (all adj. *p*s > 0.06, see Table S7) and older adults’ choice behavior (all adj. *p*s > 0.30, see Table S7).

**Theory of mind (ToM).** For both younger and older adults’ choice behavior, no significant main or interaction effects involving ToM were observed (all adj. *ps* > 0.10, see Table S7).

### **Moderation effect of empathy, compassion, and theory of mind on reaction times**

**Empathy & compassion.**

No significant main or interaction effects with respect to empathy were found on both younger or older adults’ RTs (all adj. *p*s > 0.08, see Table S8). However, we observed a significant €self × compassion and €other × compassion interaction for younger adults (see Table S8). While participants, in general, reacted quicker with higher €self (main effect €self: *F*(60.97) = 7.34, *p* = 0.009), younger adults with higher compassion scores showed less of this speeding with increasing €self (*F*(61.01) = 7.19, *p* = 0.009, adj. *p* = 0.02, see Figure S2A) and even slower RTs with higher €other (*F*(60.87) = 7.32, *p* = 0.009, adj. *p* = 0.03, see Figure S2B). We did not find any main or interaction effect involving compassion in older adults (all adj. *p*s > 0.47, see Table S8 for the full output of the model).

**Theory of mind.** A significant 2-way interaction of €self × ToM was observed for younger adults’ RT (*F*(60.90) = 7.08, *p* = 0.01, adj. *p* = 0.02). Interestingly, with lower ToM, RTs were more strongly influenced by €self (see Figure S2C). No further main or interaction effects with regard to younger adults’ ToM abilities were found (all adj. *p*s > 0.21, see Table S8). With respect to older adults’ RTs and ToM abilities, we did not observe any significant main or interaction effect (all adj. *p*s > 0.52, see Table S8).

**Figure S2**

*RTs in younger adults (YA) as a function of either €self or €other and different socio-affective measures.*

*Note.* (A) and (B) Compassion × €self/€other on YAs’ RTs. Generally, participants reacted quicker the higher €self (main effect €self: *F*(60.97) = 7.34, *p* = 0.009). However, YAs with higher compassion scores showed less of this effect of €self (i.e., less speeding with higher €self) on RTs (*F*(61.01) = 7.19, *p* = 0.009, adj. *p* = 0.02, see Figure A), and in fact descriptively, even slower RTs with higher €other (*F*(60.87) = 7.32, *p* = 0.009, adj. *p* = 0.03, see Figure B). (C) ToM accuracy × €self on YAs’ RTs. As stated above, in general, participants reacted quicker the higher €self (main effect €self: *F*(60.97) = 19.17, *p* < 0.001). However, this effect was flattened in YA who scored high in ToM (*F*(60.90) = 7.08, *p* = 0.01, adj. *p* = 0.02).

**Table S5**

*Moderator of cognitive measures regarding the interaction of €Self x €Other in younger and older adults’ choice behavior.*

| Choice Behavior | Younger Adults | | | | | |  | Older Adults | | | | | |  |
| --- | --- | --- | --- | --- | --- | --- | --- | --- | --- | --- | --- | --- | --- | --- |
| Inhibitory Control | DF | | F-value | | p-value | | d | DF | | F-value | | p-value | | d |
| €Self | 57.98 | | 320.02 | | < 0.001*** | | 0.45 | 42.00 | | 26.21 | | < 0.001*** | | 0.12 |
| €Other | 58.00 | | 190.96 | | < 0.001*** | | 0.41 | 42.02 | | 6.75 | | 0.01* | | 0.05 |
| Inhibition | 58.02 | | 1.12 | | 0.30 | | 0.11 | 41.99 | | 5.18 | | 0.03* | | 0.28 |
| €Self x €Other | 57.99 | | 58.69 | | < 0.001*** | | 0.06 | 42.11 | | 0.64 | | 0.43 | | 0.003 |
| €Self x Inhibition | 58.01 | | 1.32 | | 0.26 | | 0.04 | 42.00 | | 1.38 | | 0.25 | | 0.04 |
| €Other x Inhibition | 58.01 | | 0.14 | | 0.71 | | 0.02 | 42.00 | | 0.67 | | 0.42 | | 0.03 |
| €Self x €Other x Inhibition | 58.01 | | 0.00 | | 0.97 | | 0.0002 | 42.04 | | 6.27 | | 0.02* | | 0.01 |
| Choice Behavior | Younger Adults | | | | | |  | Older Adults | | | | | |  |
| Fluid Intelligence | DF | F-value | | p-value | | Adj. p | d | DF | F-value | | p-value | | Adj. p | d |
| €Self | 60.99 | 275.76 | | < 0.001*** | |  | 0.40 | 45.00 | 28.66 | | < 0.001*** | |  | 0.11 |
| €Other | 61.00 | 189.35 | | < 0.001*** | |  | 0.39 | 45.02 | 6.41 | | 0.02* | |  | 0.05 |
| Fluid Intelligence | 61.02 | 0.74 | | 0.39 | | 0.39 | 0.12 | 44.99 | 3.27 | | 0.08 | | 0.15 | 0.26 |
| €Self x €Other | 60.99 | 61.72 | | < 0.001*** | |  | 0.05 | 45.09 | 0.67 | | 0.42 | |  | .003 |
| €Self x Fluid | 61.01 | 0.75 | | 0.39 | | 0.39 | 0.04 | 45.01 | 3.79 | | 0.06 | | 0.08 | .08 |
| €Other x Fluid | 61.01 | 0.43 | | 0.51 | | 0.51 | 0.03 | 45.01 | 4.75 | | 0.03* | | 0.07 | .07 |
| €Self x €Other x Fluid | 61.01 | 0.02 | | 0.89 | | 0.89 | 0.001 | 45.10 | 1.81 | | 0.19 | | 0.37 | .009 |
| Verbal Intelligence | DF | F-value | | p-value | | Adj. p | d | DF | F-value | | p-value | | Adj. p | d |
| €Self | 60.99 | 292.67 | | < 0.001*** | |  | 0.42 | 45.01 | 28.29 | | < 0.001*** | |  | 0.11 |
| €Other | 61.00 | 194.02 | | < 0.001*** | |  | 0.39 | 45.03 | 5.80 | | 0.02* | |  | 0.05 |
| SAW | 60.99 | 1.43 | | 0.24 | | 0.39 | 0.14 | 44.99 | 0.33 | | 0.57 | | 0.57 | .06 |
| €Self x €Other | 60.99 | 61.86 | | < 0.001*** | |  | 0.05 | 45.10 | 0.64 | | 0.43 | |  | 0.003 |
| €Self x SAW | 60.97 | 4.54 | | 0.04* | | 0.07 | 0.08 | 44.99 | 3.16 | | 0.08 | | 0.08 | 0.05 |
| €Other x SAW | 60.99 | 1.95 | | 0.17 | | 0.34 | 0.06 | 45.01 | 0.02 | | 0.90 | | 0.90 | 0.004 |
| €Self x €Other x SAW | 60.89 | 0.17 | | 0.68 | | 0.89 | 0.004 | 45.03 | 0.01 | | 0.94 | | 0.94 | 0.0004 |

*Notes*. Fluid = Composite of fluid intelligence. SAW = Spot a word test. Adj. p = p value adjusted for the false discovery rate.

*** p < 0.001, ** p < 0.01, * p < 0.05

**Table S6**

*Moderator analyses of cognitive measures regarding the interaction of €Self x €Other in younger and older adults’ RTs.*

| Reaction Times | Younger Adults | | | | | |  | Older Adults | | | | | |  |
| --- | --- | --- | --- | --- | --- | --- | --- | --- | --- | --- | --- | --- | --- | --- |
| Inhibitory Control | DF | | F-value | | p-value | | d | DF | | F-value | | p-value | | d |
| €Self | 57.98 | | 11.57 | | 0.001** | | 0.07 | 41.94 | | 5.16 | | 0.03* | | 0.04 |
| €Other | 57.82 | | 3.70 | | 0.06 | | 0.04 | 42.05 | | 4.30 | | 0.04* | | 0.03 |
| Inhibition | 57.98 | | 1.96 | | 0.17 | | 0.21 | 41.98 | | 0.04 | | 0.84 | | 0.05 |
| Trial | 10510.24 | | 1566.59 | | < 0.001*** | | 0.01 | 7716.80 | | 873.85 | | < 0.001*** | | 0.009 |
| €Self x €Other | 57.96 | | 27.89 | | < 0.001*** | | 0.04 | 41.94 | | 0.14 | | 0.71 | | 0.002 |
| €Self x Inhibition | 58.07 | | 0.58 | | 0.45 | | 0.02 | 41.90 | | 0.13 | | 0.72 | | 0.01 |
| €Other x Inhibition | 57.86 | | 0.30 | | 0.59 | | 0.02 | 41.92 | | 0.57 | | 0.45 | | 0.02 |
| €Self x €Other x Inhibition | 57.99 | | 0.14 | | 0.71 | | 0.004 | 41.85 | | 3.50 | | 0.07 | | 0.02 |
|  | Younger Adults | | | | | |  | Older Adults | | | | | |  |
| Fluid Intelligence | DF | F-value | | p-value | | Adj. p |  | DF | F-value | | p-value | | Adj. p |  |
| €Self | 60.99 | 11.40 | | 0.001** | |  | 0.07 | 45.92 | 7.33 | | 0.01** | |  | 0.04 |
| €Other | 60.85 | 3.64 | | 0.06 | |  | 0.05 | 46.06 | 5.83 | | 0.02* | |  | 0.03 |
| Fluid Intelligence | 61.00 | 3.18 | | 0.08 | | 0.14 | 0.03 | 46.00 | 0.38 | | 0.54 | | 0.54 | 0.17 |
| Trial | 11035.87 | 1748.65 | | < 0.001*** | |  | 0.01 | 8417.66 | 1003.24 | | < 0.001*** | |  | 0.009 |
| €Self x €Other | 60.96 | 26.92 | | < 0.001*** | |  | 0.04 | 45.94 | 0.47 | | 0.50 | |  | 0.003 |
| €Self x Fluid | 61.10 | 0.32 | | 0.57 | | 0.75 | 0.02 | 45.93 | 0.02 | | 0.88 | | 0.88 | 0.005 |
| €Other x Fluid | 60.92 | 1.03 | | 0.31 | | 0.31 | 0.04 | 46.08 | 1.88 | | 0.18 | | 0.35 | 0.03 |
| €Self x €Other x Fluid | 60.99 | 0.00 | | 0.98 | | 0.98 | 0.0004 | 45.96 | 4.13 | | 0.048* | | 0.10 | 0.02 |
| Verbal Intelligence | DF | F-value | | p-value | | Adj. p |  | DF | F-value | | p-value | | Adj. p |  |
| €Self | 60.99 | 11.37 | | 0.001** | |  | 0.07 | 45.92 | 7.44 | | 0.01** | |  | 0.04 |
| €Other | 60.83 | 3.67 | | 0.06 | |  | 0.04 | 46.05 | 5.61 | | 0.02* | |  | 0.03 |
| SAW | 60.94 | 2.27 | | 0.14 | | 0.14 | 0.25 | 45.99 | 1.61 | | 0.21 | | 0.42 | 0.26 |
| Trial | 11038.96 | 1750.42 | | < 0.001*** | |  | 0.01 | 8416.33 | 1004.83 | | < 0.001*** | |  | 0.009 |
| €Self x €Other | 60.98 | 30.58 | | < 0.001*** | |  | 0.04 | 45.95 | 0.43 | | 0.51 | |  | 0.004 |
| €Self x SAW | 60.93 | 0.11 | | 0.75 | | 0.75 | 0.01 | 45.85 | 0.74 | | 0.40 | | 0.79 | 0.02 |
| €Other x SAW | 60.79 | 1.60 | | 0.21 | | 0.31 | 0.04 | 45.94 | 0.00 | | 0.98 | | 0.98 | 0.0004 |
| €Self x €Other x SAW | 60.95 | 8.29 | | 0.005** | | 0.01* | 0.03 | 45.87 | 0.54 | | 0.47 | | 0.47 | 0.006 |

*Notes.* Fluid = Composite of fluid intelligence. SAW = Spot a word test. Adj. p = p value adjusted for the false discovery rate.

*** p < 0.001, ** p < 0.01, * p < 0.05

**Table S7**

*Moderator analyses of socio-affective and -cognitive processes regarding the interaction of €Self x €Other in younger and older adults’ choice behavior.*

| Choice Behavior | Younger Adults | | | |  | Older Adults | | | |  |
| --- | --- | --- | --- | --- | --- | --- | --- | --- | --- | --- |
| Empathy | DF | F-value | p-value | Adj. p | d | DF | F-value | p-value | Adj. p | d |
| €Self | 60.99 | 283.75 | < 0.001*** |  | 0.42 | 46.01 | 27.20 | < 0.001*** |  | 0.11 |
| €Other | 61.00 | 190.83 | < 0.001*** |  | 0.39 | 46.02 | 6.77 | 0.01* |  | 0.05 |
| Empathy | 61.00 | 1.34 | 0.25 | 0.25 | 0.10 | 46.00 | 0.78 | 0.38 | 0.94 | 0.07 |
| €Self x €Other | 60.99 | 61.63 | < 0.001*** |  | 0.05 | 46.10 | 0.57 | 0.46 |  | 0.003 |
| €Self x Empathy | 60.99 | 2.30 | 0.13 | 0.20 | 0.04 | 46.00 | 0.06 | 0.81 | 0.996 | 0.006 |
| €Other x Empathy | 61.00 | 0.77 | 0.39 | 0.58 | 0.03 | 46.01 | 2.65 | 0.11 | 0.31 | 0.03 |
| €Self x €Other x Empathy | 60.99 | 0.05 | 0.83 | 0.83 | 0.001 | 46.05 | 0.08 | 0.78 | 0.78 | 0.001 |
| Compassion | DF | F-value | p-value | Adj. p | d | DF | F-value | p-value | Adj. p | d |
| €Self | 60.99 | 253.00 | < 0.001*** |  | 0.41 | 46.00 | 25.24 | < 0.001*** |  | 0.11 |
| €Other | 61.00 | 175.50 | < 0.001*** |  | 0.40 | 46.02 | 5.50 | 0.02* |  | 0.04 |
| Compassion | 61.01 | 5.86 | 0.02* | 0.06 | 0.21 | 46.00 | 0.13 | 0.72 | 0.94 | 0.02 |
| €Self x €Other | 60.99 | 62.90 | < 0.001*** |  | 0.06 | 46.07 | 0.29 | 0.59 |  | 0.002 |
| €Self x Compassion | 61.00 | 4.56 | 0.04* | 0.11 | 0.06 | 46.00 | 0.00 | 0.99 | 0.996 | 0.00009 |
| €Other x Compassion | 61.00 | 0.02 | 0.88 | 0.88 | 0.004 | 46.02 | 0.04 | 0.84 | 0.84 | 0.003 |
| €Self x €Other x Compassion | 61.00 | 1.18 | 0.28 | 0.42 | 0.009 | 46.02 | 0.48 | 0.49 | 0.74 | 0.002 |
| Theory of mind | DF | F-value | p-value | Adj. p | d | DF | F-value | p-value | Adj. p | d |
| €Self | 60.98 | 234.22 | < 0.001*** |  | 0.43 | 46.01 | 21.22 | < 0.001*** |  | 0.11 |
| €Other | 61.00 | 139.17 | < 0.001*** |  | 0.37 | 46.04 | 8.00 | 0.01** |  | 0.06 |
| ToM | 60.98 | 1.81 | 0.18 | 0.25 | 0.60 | 45.99 | 0.01 | 0.94 | 0.94 | 0.03 |
| €Self x €Other | 60.99 | 39.28 | < 0.001*** |  | 0.05 | 46.13 | 1.41 | 0.24 |  | 0.005 |
| €Self x ToM | 60.97 | 1.10 | 0.30 | 0.30 | 0.15 | 46.00 | 0.08 | 0.78 | 0.996 | 0.03 |
| €Other x ToM | 60.99 | 2.11 | 0.15 | 0.46 | 0.23 | 46.02 | 1.64 | 0.21 | 0.31 | 0.12 |
| €Self x €Other x ToM | 60.98 | 4.74 | 0.03* | 0.10 | 0.08 | 46.04 | 1.09 | 0.30 | 0.74 | 0.02 |

*Notes.* ToM = Theory of mind. Adj. p = p value adjusted for the false discovery rate.

*** p < 0.001, ** p < 0.01, * p < 0.05

**Table S8**

*Moderator analyses of socio-affective and -cognitive processes regarding the interaction of €Self x €Other in younger and older adults’ reaction times.*

| Reaction Times | Younger Adults | | | |  | Older Adults | | | |  |
| --- | --- | --- | --- | --- | --- | --- | --- | --- | --- | --- |
| Empathy | DF | F-value | p-value | Adj. p | d | DF | F-value | p-value | Adj. p | d |
| €Self | 60.98 | 12.21 | <0.001*** |  | 0.07 | 45.93 | 7.28 | 0.01** |  | 0.04 |
| €Other | 60.84 | 3.84 | 0.06 |  | 0.04 | 46.06 | 5.57 | 0.02* |  | 0.03 |
| Empathy | 60.97 | 1.30 | 0.26 | 0.50 | 0.14 | 46.00 | 0.00 | 0.97 | 0.97 | 0.006 |
| Trial | 11033.92 | 1747.78 | < 0.001*** |  | 0.13 | 8415.42 | 1003.76 | < 0.001*** |  | 0.009 |
| €Self x €Other | 60.97 | 27.98 | < 0.001*** |  | 0.04 | 45.95 | 0.40 | 0.53 |  | 0.003 |
| €Self x Empathy | 61.01 | 3.17 | 0.08 | 0.08 | 0.04 | 45.91 | 0.14 | 0.71 | 0.84 | 0.006 |
| €Other x Empathy | 60.85 | 2.21 | 0.14 | 0.21 | 0.04 | 46.01 | 0.53 | 0.47 | 0.71 | 0.01 |
| €Self x €Other x Empathy | 60.98 | 1.69 | 0.20 | 0.30 | 0.01 | 45.89 | 0.94 | 0.94 | 0.50 | 0.005 |
| Compassion | DF | F-value | p-value | Adj. p | d | DF | F-value | p-value | Adj. p | d |
| €Self | 60.97 | 7.34 | 0.01** |  | 0.05 | 45.91 | 7.21 | 0.01* |  | 0.04 |
| €Other | 60.85 | 1.44 | 0.23 |  | 0.03 | 46.02 | 4.13 | 0.048* |  | 0.03 |
| Compassion | 60.97 | 0.00 | 0.98 | 0.98 | 0.004 | 46.00 | 2.07 | 0.16 | 0.47 | 0.17 |
| Trial | 11037.45 | 1749.30 | < 0.001*** |  | 0.01 | 8415.31 | 1004.50 | < 0.001*** |  | 0.009 |
| €Self x €Other | 60.97 | 21.99 | < 0.001*** |  | 0.04 | 45.92 | 0.33 | 0.57 |  | 0.003 |
| €Self x Compassion | 61.01 | 7.19 | 0.01** | 0.02* | 0.06 | 45.89 | 0.08 | 0.78 | 0.84 | 0.004 |
| €Other x Compassion | 60.87 | 7.32 | 0.01** | 0.03* | 0.06 | 46.00 | 1.14 | 0.29 | 0.71 | 0.01 |
| €Self x €Other x Compassion | 60.99 | 2.17 | 0.15 | 0.30 | 0.01 | 45.86 | 0.05 | 0.82 | 0.82 | 0.001 |
| Theory of mind | DF | F-value | p-value | Adj. p | d | DF | F-value | p-value | Adj. p | d |
| €Self | 60.97 | 19.17 | < 0.001*** |  | 0.09 | 45.95 | 4.89 | 0.03* |  | 0.04 |
| €Other | 60.80 | 5.23 | 0.03* |  | 0.06 | 46.12 | 3.60 | 0.06 |  | 0.03 |
| ToM | 60.94 | 0.94 | 0.34 | 0.50 | 0.60 | 46.00 | 0.04 | 0.84 | 0.97 | 0.15 |
| Trial | 11035.93 | 1750.22 | < 0.001*** |  | 0.01 | 8417.12 | 1003.92 | < 0.001*** |  | 0.009 |
| €Self x €Other | 60.96 | 26.12 | < 0.001*** |  | 0.05 | 45.99 | 0.00 | 0.96 |  | 0.0003 |
| €Self x ToM | 60.90 | 7.08 | 0.01** | 0.02* | 0.29 | 45.88 | 0.04 | 0.84 | 0.84 | 0.02 |
| €Other x ToM | 60.77 | 1.63 | 0.21 | 0.21 | 0.15 | 46.01 | 0.06 | 0.80 | 0.80 | 0.02 |
| €Self x €Other x ToM | 60.94 | 0.99 | 0.32 | 0.32 | 0.05 | 45.86 | 1.42 | 0.24 | 0.50 | 0.03 |

*Notes.* ToM = Theory of mind. Adj. p = p value adjusted for the false discovery rate.

*** p < 0.001, ** p < 0.01, * p < 0.05

**Figure S3.**

*Histograms of cognitive variables.*

**Figure S4.**

*Histogram of EmpaToM Variables.*

## **Results of the HDDM analyses**

We here report the results of the DDM analysis for the initial bias (*z*-parameter) and the amount of relative evidence accumulated (*a*-parameter). As our research question focusses on prosocial decision behavior, the other-serving condition was included as the intercept of the regression model. This modeling approach enables conclusions regarding the initial bias or the amount of relative evidence toward accepting an other-serving offer, indicating a prosocial decision. As a result, the regression coefficients for the self-serving and rational condition can be interpreted relative to the other-serving condition.

### ***Initital Bias (z)***

Next, we tested whether the initial bias (*z*-parameter) differed as a function of condition (other-serving vs. selfserving vs. rational). Estimates of the initial bias (*z)* can range from 0 to 1, with values > 0.50 representing a bias towards the upper boundary (acceptance of the offer type) and values < 0.50 reflecting an initial bias towards the lower boundary (rejection of the offer type).

In younger adults, we observed different initial biases for the three different conditions (other-serving vs. self-serving vs. rational, see **Figures S5A** and **S5B**). There was no initial bias for the intercept, i.e., other-serving condition (*z*_YA-other-serving_ = 0.49, probability(*z*_YA-other-serving_ < 0.50) = 0.77). However, compared to the other-serving condition, YA showed a meaningfully increased initial bias in the rational condition towards the acceptance of the offer (*β-z*_YA-rational_ = 0.14, probability(*β-z*_YA-rational_ > 0) = 1.0, *z*_YA-rational_ = 0.63). Further, they showed a meaningfully decreased initial bias for the self-serving condition (i.e., towards the rejection of the offer type (*β-z*_YA-self-serving_ = -0.03, probability(*β-z*_YA-self-serving_ < 0) = 0.96, *z*_YA-self-serving_ = 0.46), compared to the other-serving condition.

In older adults, we also observed meaningful differences in initial biases depending on the condition (see **Figures S5A** and **S5B**). They did show an initial bias towards the lower boundary, i.e., the rejection of the offer type for the other-serving condition (intercept; *z*_OA-other-serving_ = 0.47, probability(*z*_OA-other-serving_ < 0.5) = 0.98). Further, they showed higher initial biases in both the rational (*β-z*_OA-rational_ = 0.08, *z*_OA-rational_ = 0.55, probability(*β*-z_OA-rational_ > 0) = 1.0) and self-serving condition (*β-z*_OA-self-serving_ = 0.06, *z*_OA-self-serving_ = 0.53, probability(*β*-z_OA-self-serving_ > 0) = 1.0), compared to the other-serving condition.

We did not observe age group differences between younger and older adults with respect to their initial bias in the other-serving condition (i.e., the intercept; probability(z_YA-other-serving_ > z_OA-other-serving_) = 0.86). However, the beta weights for the initial bias in the self-serving condition were significantly higher in older compared to younger adults (probability(*β*-z_OA-self-serving_ > *β*-z_YA-self-serving_) = 1.0). Also, the beta weights for the initial bias in the rational condition varied meaningfully between age groups. That is, both age groups did show increased initial biases for the rational compared to the other-serving condition, but this effect was more pronounced in younger compared to older adults (probability(*β*-z_YA-rational_ > *β*-z_OA-rational_) = 0.98).

This means that older adults had a larger initial bias towards accepting self-serving offers than younger adults. Younger adults, on the other hand, had a larger bias towards accepting rational offers than older adults.

In an additional analysis, we tested whether the different z-parameter regression weights were linked to individual Fehr-Schmidt preferences as derived from a Fehr-Schmidt model (Fehr and Schmidt 1999). These pairwise spearman correlations showed that, the higher an individual’s Fehr-Schmidt preference, the lower this person’s bias towards accepting self-serving decision options (rho = -.32, S = 154465, p = .003, BF = 13). Individual Fehr-Schmidt preferences were not significantly related to other DDM parameters (see **Figure S4** for visualization).

**Figure S4**. Pairwise correlation between individual Fehr-Schmidt preferences and the different principal individual DDM parameters of interest. The correlation coefficients with absolute values above .3 are statistically significant at p<.05. v = v_self×other_, z_gen = z_other-serving_, z_self = z_self-serving_, z_rat = z_rational_, fs_pref = Fehr-Schmidt preference.

**Figure S5.** *Posterior density plots with respect to the initital bias (z-parameter)*

*Note.* Posterior density plots concerning the initital bias (*z*-parameter). (A) Initial bias in younger adults (YA; purple) and older adults (OA; green) of the condition intercept (other-serving condition). The grey dotted vertical line (x = 0.50) indicates no choice bias. Values > 0.50 represent a bias towards the upper boundary (accepting the offer type). Values < 0.50 represent a bias towards the lower boundary (rejecting the offer type). (B) Beta weights correspond to the within-subject effects of the self-serving and rational condition on the z-parameter in YA (red and orange) and OA (pink and grey). Beta weights illustrate how the initial bias changes in self-serving and rational conditions with respect to the intercept (other-serving condition).

### ***Boundary separation (a)***

Finally, we investigated whether the amount of relative evidence that participants required to cross a decision boundary (*a*-parameter, cf. Figure 1C) and, thus, reach a decision differed depending on the condition (other-serving vs. self-serving vs. rational). Due to convergence problems in the initial model fitting procedure, in the estimation process of the *a*-parameters in older adults, the following results must be interpreted cautiously.

In younger adults, the boundary separation in the other-serving condition (intercept) was *a*_YA-other-serving_ = 2.31 (see **Figure S6A**). Compared to this intercept, the boundary separation decreased in the self-serving condition (*β-a*_YA-self-serving_ = -0.25, *a*_YA-self-serving_ = 2.06, probability(*β-a*_YA-self-serving_ < 0) = 1.0, see **Figure S6B**). This indicates that less evidence had to be accumulated in the self-serving (compared to the other-serving) condition to reach a decision. No meaningful change in boundary separation was observed for the rational condition compared to the intercept, i.e., other-serving condition (*β-a*_YA-rational_ = -0.04, *a*_YA-rational_ = 2.27, probability(*β-a*_YA-rational_ < 0) = 0.81, see **Figure S6B**).

Older adults showed a boundary separation of *a*_OA-other-serving_ = 2.29 (see Figure S4A) in the other-serving condition (intercept). However, the boundary separation decreased in both the self-serving (*β-a*_OA-self-serving_ = -0.26, *a*_OA-self-serving_ = 2.03, probability(*β-a*_OA-self-serving_ < 0) = 1.0) and rational condition (*β-a*_OA-rational_ = -0.18, *a*_OA-rational_ = 2.12, probability(*β-a*_OA-rational_ < 0) = 1.0), as compared to the other-serving condition (see Figure S4B). This suggests that older adults had to accumulate less evidence to reach a decision in the self-serving and rational condition, as compared to the other-serving condition.

No age group difference concerning the other-serving condition in boundary separation was observed (probability(*a*_YA-other-serving_ > *a*_OA-other-serving_) = 0.55). Moreover, we did not find age differences in boundary separation when considering the beta weights for the self-serving condition in younger compared to older adults (probability(*β-a*_YA-self-serving_ > *β-a*_OA-self-serving_) = 0.56). However, the beta-weights for the rational condition varied meaningfully between age groups (probability(*β-a*_YA-rational_ > *β-a*_OA-rational_) = 0.98). Thus, in the rational (relative to the other-serving) condition, older adults showed a larger decrease in boundary separation than younger adults.

**Figure S6.** *Posterior density plots with respect to the boundary separation (a-parameter)*

*Note.* Posterior density plots concerning the boundary separation (*a*-parameter). (A) Boundary separation in younger adults (YA; purple) and older adults (OA; green) with respect to the intercept, i.e., other-serving condition. (B) Beta weights corresponding to the within-subject effects of the self-serving and rational condition on boundary separation in YA (red and orange) and OA (pink and grey). Beta weights illustrate how boundary separation changes in self-serving and rational conditions relative to the intercept (other-serving condition).

**Moderation Analyses on HDDM parameters**

**V-Parameter: Drift Rate**

The mean group posteriors of the drift rate (based on the interaction of €self × €other) were not significantly predicted by any of the pre-defined moderators (all (adj.) *p*s > 0.15, see Table S9).

**Z-Parameter: Initial Bias**

The mean group posteriors of the initial biases (separately for other-serving, self-serving, and rational conditions) were not significantly predicted by any of the pre-defined moderators (all (adj.) *p*s > 0.09, see Table S10).

**A-Parameter: Boundary Separation**

In younger adults, the mean group posteriors of boundary separation for the other-serving and rational condition were significantly predicted by ToM-abilities (other-serving: *b* = -0.83, adj. *p* = 0.048; rational: *b* = -0.84, adj. *p* = 0.048, see Table S11). Both effects showed a negative association, indicating that younger adults with higher ToM-abilities need to accumulate less evidence to reach a decision in the other-serving and rational condition (i.e., reduced boundary separation). No further significant associations between boundary separation and the pre-defined moderators were observed with respect to younger adults, irrespective of the condition (all adj. *ps* > 0.45, see Table S12). In older adults, across all three conditions (other-serving, self-serving, and rational), significant negative associations were found between boundary separation and fluid abilities, as well as between boundary separation and verbal abilities (-0.35 > *b* > -0.24, all adj. *p*s < 0.046). All effects showed a negative relationship, illustrating that older adults with better cognitive functioning showed reduced boundary separation (in all three conditions), i.e., they had to accumulate less evidence to reach a decision.

**Table S9**

*Linear models predicting drift rates (v-parameter). Inhibitory control, fluid abilities, verbal abilities, empathy, compassion, and theory of mind predicting drift rate of €Self x €Other*

|  | Younger adults | | | | | | | Older adults | | | | | | |
| --- | --- | --- | --- | --- | --- | --- | --- | --- | --- | --- | --- | --- | --- | --- |
|  | b | SE | DF | t | p | adj. p | r | b | SE | DF | t | p | adj. p | r |
| Inhibition | 0.01 | 0.01 | 58 | 0.75 | 0.46 |  | 0.10 | 0.01 | 0.01 | 42 | 1.35 | 0.19 |  | 0.20 |
| Fluid intelligence | -0.002 | 0.01 | 61 | -0.21 | 0.83 | 0.83 | -0.03 | 0.01 | 0.01 | 46 | 1.82 | 0.08 | 0.15 | 0.26 |
| Verbal intelligence | 0.004 | 0.01 | 61 | 0.47 | 0.64 | 0.83 | 0.06 | -0.0003 | 0.01 | 46 | -0.05 | 0.96 | 0.96 | -0.01 |
| Empathy | 0.003 | 0.01 | 61 | 0.45 | 0.66 | 0.78 | 0.06 | -0.01 | 0.004 | 46 | -1.16 | 0.25 | 0.43 | -0.17 |
| Compassion | -0.002 | 0.01 | 61 | -0.28 | 0.78 | 0.78 | -0.04 | -0.001 | 0.003 | 46 | 0.14 | 0.89 | 0.89 | 0.02 |
| Theory of mind | 0.02 | 0.03 | 61 | 0.74 | 0.46 | 0.78 | 0.09 | 0.02 | 0.02 | 46 | 1.07 | 0.29 | 0.43 | 0.16 |

*Note.* Fluid intelligence = Composite of fluid intelligence. Verbal Intelligence = Composite of verbal intelligence.

* p < 0.05

**Table S10**

*Linear models predicting initial bias (z-parameter). Inhibitory control, fluid abilities, verbal abilities, empathy, compassion, and theory of mind predicting the initial starting bias, separately for other-serving, self-serving, and rational choice options.*

|  | | Younger Adults | | | | | | | Older Adults | | | | | | |
| --- | --- | --- | --- | --- | --- | --- | --- | --- | --- | --- | --- | --- | --- | --- | --- |
|  |  | b | SE | DF | t | p | adj. p | r | b | SE | DF | t | p | adj. p | r |
| Other-serving | Inhibition | 0.002 | 0.01 | 58 | 0.20 | 0.84 |  | 0.03 | 0.01 | 0.02 | 42 | 0.51 | 0.61 |  | 0.08 |
| Other-serving | Fluid int. | -0.004 | 0.01 | 61 | -0.27 | 0.79 | 0.79 | -0.04 | 0.02 | 0.02 | 46 | 1.22 | 0.23 | 0.46 | 0.18 |
| Other-serving | Verbal Int. | 0.003 | 0.01 | 61 | 0.28 | 0.78 | 0.79 | 0.04 | 0.001 | 0.01 | 46 | 0.07 | 0.94 | 0.94 | 0.01 |
| Other-serving | Empathy | 0.01 | 0.01 | 61 | 0.79 | 0.43 | 0.43 | 0.10 | 0.01 | 0.01 | 46 | 1.10 | 0.28 | 0.73 | 0.16 |
| Other-serving | Compassion | -0.01 | 0.01 | 61 | -0.94 | 0.35 | 0.43 | -0.12 | -0.003 | 0.01 | 46 | -0.34 | 0.73 | 0.73 | -0.05 |
| Other-serving | ToM | -0.07 | 0.05 | 61 | -1.48 | 0.14 | 0.43 | -0.19 | -0.03 | 0.05 | 46 | -0.51 | 0.61 | 0.73 | -0.07 |
| Self-serving | Inhibition | -0.02 | 0.02 | 58 | -1.16 | 0.25 |  | -0.15 | 0.003 | 0.03 | 42 | 0.12 | 0.91 |  | 0.02 |
| Self-serving | Fluid int. | 0.001 | 0.02 | 61 | 0.04 | 0.97 | 0.97 | 0 | 0.02 | 0.03 | 46 | 0.78 | 0.44 | 0.63 | 0.11 |
| Self-serving | Verbal Int. | 0.04 | 0.02 | 61 | 2.06 | 0.04* | 0.09 | 0.26 | 0.01 | 0.02 | 46 | 0.49 | 0.63 | 0.63 | 0.07 |
| Self-serving | Empathy | 0.02 | 0.01 | 61 | 1.11 | 0.27 | 0.27 | 0.15 | 0.02 | 0.02 | 46 | 1.26 | 0.22 | 0.47 | 0.18 |
| Self-serving | Compassion | -0.02 | 0.01 | 61 | -1.20 | 0.24 | 0.27 | -0.15 | -0.01 | 0.01 | 46 | -0.41 | 0.69 | 0.69 | -0.06 |
| Self-serving | ToM | -0.11 | 0.07 | 61 | -1.49 | 0.14 | 0.27 | -0.19 | -0.08 | 0.08 | 46 | -1.02 | 0.31 | 0.47 | -0.15 |
| Rational | Inhibition | -0.01 | 0.03 | 58 | -0.29 | 0.77 |  | -0.04 | 0.02 | 0.02 | 42 | 0.88 | 0.38 |  | 0.14 |
| Rational | Fluid int. | 0.02 | 0.03 | 61 | 0.46 | 0.65 | 0.65 | 0.06 | 0.03 | 0.02 | 46 | 1.26 | 0.22 | 0.43 | 0.18 |
| Rational | Verbal In. | -0.03 | 0.03 | 61 | -0.99 | 0.33 | 0.65 | -0.13 | 0.01 | 0.02 | 46 | 0.58 | 0.56 | 0.56 | 0.09 |
| Rational | Empathy | 0.02 | 0.02 | 61 | 1.16 | 0.25 | 0.74 | 0.15 | 0.01 | 0.01 | 46 | 0.86 | 0.40 | 0.74 | 0.13 |
| Rational | Compassion | -0.01 | 0.02 | 61 | -0.34 | 0.74 | 0.74 | -0.04 | 0.002 | 0.01 | 46 | 0.21 | 0.84 | 0.84 | 0.03 |
| Rational | ToM | 0.07 | 0.11 | 61 | 0.65 | 0.52 | 0.74 | 0.08 | 0.04 | 0.06 | 46 | 0.69 | 0.49 | 0.74 | 0.10 |

*Note.* Fluid int. = Composite of fluid intelligence. Verbal Int. = Composite of verbal intelligence. ToM = Theory of mind.

* p < 0.05

**Table S11.** Linear models predicting boundary separation (a-parameter). Inhibitory control, fluid abilities, verbal abilities, empathy, compassion, and theory of mind predicting boundary separation, separately for other-serving, self-serving, and rational choice options.

|  | | Younger Adults | | | | | | | Older Adults | | | | | | |
| --- | --- | --- | --- | --- | --- | --- | --- | --- | --- | --- | --- | --- | --- | --- | --- |
|  |  | b | SE | DF | t | p | adj. p | r | b | SE | DF | t | p | adj. p | r |
| Other-serving | Inhibition | 0.001 | 0.08 | 58 | 0.01 | 0.99 |  | 0 | -0.25 | 0.14 | 42 | -1.80 | 0.09 |  | -0.27 |
| Other-serving | Fluid int. | -0.08 | 0.11 | 61 | -0.76 | 0.45 | 0.45 | -0.10 | -0.35 | 0.15 | 46 | -2.28 | 0.03* | 0.045* | -0.32 |
| Other-serving | Verbal int. | -0.08 | 0.09 | 61 | -0.83 | 0.41 | 0.45 | -0.10 | -0.24 | 0.12 | 46 | -2.06 | 0.045* | 0.045* | -0.29 |
| Other-serving | Empathy | 0.03 | 0.07 | 61 | 0.49 | 0.63 | 0.63 | 0.06 | 0.02 | 0.09 | 46 | -0.23 | 0.82 | 0.82 | -0.03 |
| Other-serving | Compassion | -0.05 | 0.07 | 61 | -0.73 | 0.47 | 0.63 | -0.09 | 0.08 | 0.07 | 46 | 1.12 | 0.27 | 0.40 | 0.16 |
| Other-serving | ToM | -0.83 | 0.34 | 61 | -2.47 | 0.02* | 0.048* | -0.30 | 0.55 | 0.42 | 46 | 1.33 | 0.19 | 0.40 | 0.19 |
| Self-serving | Inhibition | -0.01 | 0.08 | 58 | -0.15 | 0.88 |  | -0.02 | -0.27 | 0.14 | 42 | -1.95 | 0.06 |  | -0.29 |
| Self-serving | Fluid int. | -0.01 | 0.10 | 61 | -0.19 | 0.85 | 0.85 | -0.02 | -0.35 | 0.16 | 46 | -2.27 | 0.03* | 0.03* | -0.32 |
| Self-serving | Verbal Int. | -0.02 | 0.08 | 61 | -0.28 | 0.78 | 0.85 | -0.04 | -0.26 | 0.12 | 46 | -2.25 | 0.03* | 0.03* | -0.32 |
| Self-serving | Empathy | -0.001 | 0.06 | 61 | -0.02 | 0.99 | 0.99 | 0 | -0.04 | 0.09 | 46 | -0.41 | 0.69 | 0.69 | -0.06 |
| Self-serving | Compassion | -0.03 | 0.06 | 61 | -0.45 | 0.65 | 0.98 | -0.06 | 0.09 | 0.07 | 46 | 1.25 | 0.22 | 0.33 | 0.18 |
| Self-serving | ToM | -0.41 | 0.31 | 61 | -1.29 | 0.20 | 0.60 | -0.16 | 0.065 | 0.42 | 46 | 1.55 | 0.13 | 0.33 | 0.22 |
| Rational | Inhibition | 0.01 | 0.09 | 58 | 0.07 | 0.94 |  | 0.01 | -0.25 | 0.14 | 42 | -1.81 | 0.08 |  | -0.27 |
| Rational | Fluid int. | -0.08 | 0.11 | 61 | -0.74 | 0.46 | 0.46 | -0.09 | -0.35 | 0.15 | 46 | -2.30 | 0.03* | 0.046* | -0.32 |
| Rational | Verbal int. | -0.08 | 0.11 | 61 | -0.87 | 0.39 | 0.46 | -0.11 | -0.24 | 0.12 | 46 | -2.05 | 0.046* | 0.046* | -0.29 |
| Rational | Empathy | 0.04 | 0.07 | 61 | 0.57 | 0.57 | 0.57 | 0.07 | -0.02 | 0.09 | 46 | -0.22 | 0.83 | 0.83 | -0.03 |
| Rational | Compassion | -0.05 | 0.07 | 61 | -0.77 | 0.45 | 0.57 | -0.10 | 0.08 | 0.07 | 46 | 1.11 | 0.27 | 0.41 | 0.16 |
| Rational | ToM | -0.84 | 0.34 | 61 | -2.47 | 0.02* | 0.048* | -0.30 | 0.55 | 0.42 | 46 | 1.32 | 0.20 | 0.41 | 0.19 |

*Note.* Fluid int. = Composite of fluid intelligence. Verbal Intelligence = Composite of verbal intelligence. ToM = Theory of mind.

* p < 0.05

**Table S12**

*Posterior Predictive Checks – Younger Adults*

| stat | observed | mean | std | SEM | MSE | credible | quantile | mahalanobis |
| --- | --- | --- | --- | --- | --- | --- | --- | --- |
| accuracy | 0.41484055 | 0.410117 | 0.19519164 | 2.231191e-05 | 0.038122084 | True | 53.850792 | 0.024199544 |
| mean_ub | 1.6124573 | 1.6109482 | 0.30236858 | 2.2774611e-06 | 0.09142904 | True | 49.764 | 0.0049910145 |
| std_ub | 0.60123223 | 0.61172855 | 0.2601872 | 0.00011017308 | 0.067807555 | True | 53.88271 | 0.040341478 |
| 10q_ub | 0.9433377 | 1.0585057 | 0.21848434 | 0.013263663 | 0.060999066 | True | 30.301952 | 0.5271223 |
| 30q_ub | 1.2328377 | 1.2707397 | 0.23328803 | 0.0014365626 | 0.055859867 | True | 44.707745 | 0.16246875 |
| 50q_ub | 1.4964293 | 1.4579648 | 0.26016888 | 0.0014795191 | 0.06916737 | True | 58.01112 | 0.14784442 |
| 70q_ub | 1.8383183 | 1.7104921 | 0.329158 | 0.016339546 | 0.12468455 | True | 68.682915 | 0.38834307 |
| 90q_ub | 2.4429584 | 2.3209476 | 0.56425464 | 0.014886592 | 0.33326986 | True | 61.70309 | 0.21623327 |
| mean_lb | -1.586347 | -1.6693641 | 0.3630149 | 0.0068918355 | 0.13867165 | True | 58.92381 | 0.2286878 |
| std_lb | 0.6136045 | 0.6024849 | 0.24526753 | 0.000123646 | 0.0602798 | True | 57.444443 | 0.04533671 |
| 10q_lb | 0.9228095 | 1.1009092 | 0.24234481 | 0.03171952 | 0.09045053 | True | 23.396826 | 0.73490226 |
| 30q_lb | 1.2040224 | 1.3286825 | 0.2685924 | 0.015540156 | 0.087682046 | True | 35.120636 | 0.46412396 |
| 50q_lb | 1.4697796 | 1.5287895 | 0.30662003 | 0.0034821704 | 0.09749804 | True | 43.368256 | 0.19245291 |
| 70q_lb | 1.8133596 | 1.7916979 | 0.3905396 | 0.00046922953 | 0.15299039 | True | 51.231747 | 0.055466097 |
| 90q_lb | 2.4345248 | 2.3836374 | 0.62667656 | 0.0025895282 | 0.39531305 | True | 53.698414 | 0.08120203 |

*Note.* Quantile comparison of the observed reaction time data with reaction time data simulated based on the drift diffusion model (500 simulations), as well as the standard deviation (std), standard error of means (SEM) and mean squared error (MSE) of the simulated data. The column *credible* indicates whether the data fall within the 95 % credible interval (if “True” the model is a 95% credible fit for the observed data). *Quantile* corresponds to the posterior predictive quantile and mahalanobis indicates the *mahalanobis* distance between observed and estimated reaction time indices. *ub* = upper boundary, *lb* = lower boundary.

**Table S13**

*Posterior Predictive Checks – Older Adults*

| stat | observed | mean | std | SEM | MSE | credible | quantile | mahalanobis |
| --- | --- | --- | --- | --- | --- | --- | --- | --- |
| accuracy | 0.37532192 | 0.3800905 | 0.21723017 | 2.2739225e-05 | 0.047211684 | True | 45.0625 | 0.021951675 |
| mean_ub | 1.6421566 | 1.6395155 | 0.40381032 | 6.975271e-06 | 0.16306974 | True | 55.711765 | 0.0065403823 |
| std_ub | 0.6212577 | 0.56267816 | 0.2571273 | 0.0034315633 | 0.06954602 | True | 61.78744 | 0.22782312 |
| 10q_ub | 0.94824 | 1.1116843 | 0.30703983 | 0.02671405 | 0.120987535 | True | 32.548256 | 0.5323229 |
| 30q_ub | 1.2287391 | 1.3163218 | 0.32355967 | 0.007670724 | 0.112361595 | True | 43.89042 | 0.27068475 |
| 50q_ub | 1.5283384 | 1.5019706 | 0.36238122 | 0.00069525937 | 0.13201539 | True | 57.049862 | 0.07276252 |
| 70q_ub | 1.8834026 | 1.7576022 | 0.44115078 | 0.015825745 | 0.21043976 | True | 65.41747 | 0.2851642 |
| 90q_ub | 2.549847 | 2.3188686 | 0.6628944 | 0.053350966 | 0.4927799 | True | 67.02681 | 0.348439 |
| mean_lb | -1.5923958 | -1.6502684 | 0.4032613 | 0.0033492476 | 0.16596892 | True | 53.595833 | 0.14351162 |
| std_lb | 0.6436081 | 0.57481575 | 0.22263516 | 0.0047323904 | 0.054298796 | True | 66.075 | 0.3089915 |
| 10q_lb | 0.8442104 | 1.0951886 | 0.2980469 | 0.06299008 | 0.15182203 | True | 21.966667 | 0.84207636 |
| 30q_lb | 1.1958085 | 1.3168449 | 0.32065377 | 0.014649815 | 0.11746865 | True | 37.016666 | 0.37746763 |
| 50q_lb | 1.4852326 | 1.5161709 | 0.3575476 | 0.0009571777 | 0.12879747 | True | 48.425 | 0.08652914 |
| 70q_lb | 1.8397444 | 1.7826067 | 0.4324137 | 0.0032647257 | 0.19024634 | True | 56.320835 | 0.13213684 |
| 90q_lb | 2.5187798 | 2.3532455 | 0.6390194 | 0.027401624 | 0.43574736 | True | 63.045834 | 0.25904432 |

*Note.* Quantile comparison of the observed reaction time data with reaction time data simulated based on the drift diffusion model (500 simulations), as well as the standard deviation (std), standard error of means (SEM) and mean squared error (MSE) of the simulated data. The column *credible* indicates whether the data fall within the 95 % credible interval (if “True” the model is a 95% credible fit for the observed data). *Quantile* corresponds to the posterior predictive quantile and mahalanobis indicates the *mahalanobis* distance between observed and estimated reaction time indices. *ub* = upper boundary, *lb* = lower boundary.

***Figure S7.***

*Posterior predictive plots final model younger adults*

**Figures S6.**

*Posterior predictive plots final model older adults*

# Task instructions

**Welcome!**

This is a study on decision-making. We want to understand how people make decisions that affect not only themselves but also others. For your participation, you will receive at least €8.50 per hour. Depending on the decisions you make during the task, you can earn an additional €0 to €10.

In the room next door, another person is participating in the experiment, whom you should consider as your partner. Depending on your decisions and the outcomes of some random events, you can earn this person between €0 and €10 in addition to their wage.

You are playing with a real person, and the decisions you make can have a significant impact on their payment. Just like you, we have recruited the other person from our participant database. Since your decisions can greatly influence this person's payment, please think carefully about this person during the experiment.

You will not be told who the person is, and the other person will not learn your name or any other information about you. This means that all decisions you make during the experiment remain anonymous. Your name will not be associated with the decisions you make. Neither your partner nor the experimenter will know how you decided.

Even though your decisions are anonymous, don't forget: they significantly influence the other person's payment! The decisions you make involve real money, usually quite large sums, so please think carefully about each decision.

In the next section, we will explain the exact instructions for the task you will complete. Please read the instructions carefully. Understanding them is crucial for your ability to make good decisions – and possibly earn more money!

**How the experiment works:**

Each round begins with a cross (+) in the center of the screen. Please fixate on this cross when it appears.

After the cross appears, you will see two offers. One is on the left side of the screen, and the other is on the right side of the screen. These two offers each show an amount of money that you and your partner will win if you choose that offer. You can choose either the left or right offer.

In one of the two offers, the amount of money that you and your partner can win changes in each round. We call this the proposed allocation. The amounts for you and your partner range between €0 and €10.

In the other offer, the amount of money that you and your partner can win is always equal in each round. You and your partner each receive €5. We call this the standard allocation.

So in each round, you can choose between the proposed allocation and the standard allocation.

**Example:** If we offer you a proposed allocation of €7.50 for you and €2.50 for your partner, and you accept this offer, it would show that you prefer €7.50 (which is €2.50 above the standard of €5) for yourself and €2.50 for the other person (which is €2.50 below the standard) compared to the standard allocation (€5 for both).

When we ask you to choose between the proposed and standard allocation, we also want to get an impression of how strongly your feelings are about this decision.

You should indicate your preference towards our offers on a four-point scale:

1 = Strong preference for the left offer: Indicates that you clearly prefer the left offer and do not want to receive the right offer at all. 2 = Preference for the left offer: Means that you rather prefer the left offer over the right offer. 3 = Preference for the right offer: Means that you rather prefer the right offer over the left offer. 4 = Strong preference for the right offer: Indicates that you clearly prefer the right offer and do not want to receive the left offer at all.

**Important:** Choosing "1" or "2" will be considered a preference for the left offer; "3" or "4" as a preference for the right offer. So in addition to choosing between acceptance or rejection of an offer, you should also indicate how much more or less you prefer one allocation over another.

Another note: You must make your decision within 5 seconds after seeing both offers. If no decision is made within this time frame, both you and your partner will receive €5 for that round. Therefore, it is advantageous for you to decide promptly according to your preferences.

**How do your decisions in individual rounds contribute to your total earnings at the end?** At the end of the experiment, we randomly select ONE round from all those played by you. The result of this round will then be paid out in cash to both participants. For this reason, you should behave in each round as if it determines how much money both of you will receive at the end.

Once you've made your decision, it will be implemented with a certain probability; that is, your result does not always match with what you've chosen. In each round, 80% of time you'll get paid according to what you've chosen; in 20% of cases we'll register what you've rejected instead.

For example: If you reject an offer where both get €2.50 (which is €2.50 less than standard), you'll have an 80% chance of getting standard (€5 per person) & 20% chance getting rejected option (€2.50).

You'll know which option was registered immediately after deciding:

Green checkmark indicates implementation.

Red cross indicates non-implementation.

You'll see amounts you'd get if selected round was chosen at end.

Remember: Even though implementation probability exists best strategy still choosing preferred option since most times it'll be implemented & more often chosen higher likelihood receiving it.

Don't let this part confuse: If preferring standard allocation accept it; if preferring proposed allocation accept it too.

Keep in mind implementation probability isn't influenced by co-player's decisions; whether chosen option or other gets implemented determined by computer lottery; co-player can't influence whether decision gets implemented or not; at end must accept computer's random selection outcome regardless.

You'll now play some practice rounds which don't count but help get timing/task feel; if questions ask experimenter now otherwise start practice rounds now.

**Privacy:** As explained above making decisions about money distribution between yourself & another person; important all decisions remain secret/anonymous; other person won't know choices made; only learns which round selected for payout options available/result; knows choice had 80% chance being calculated but doesn't learn actual choice made.

To ensure true anonymity use computer program randomly select final payout round; experimenter gives both participants amount in envelope without knowing specific decision made during selected round; you'll sign payout confirmation which doesn't need readable signature – those needing confirmation know nothing about experiment/choices available; co-player also signs confirmation upon receiving payout envelope.

These measures ensure no one involved learns about choices made; co-player won't know who participant was nor get info linking choices to participant identity; data encrypted with anonymous code preventing personal info linkage; previous sections explained security measures protecting anonymity.

This experiment yields valuable results only if convinced anonymity/confidentiality maintained hence seriousness taken; ethically/legally bound adhering protocol ensuring no deception used – no surprises/tricks involved – standing by word given here.

**If you have any questions about the experiment, you now have the opportunity to ask the experimenter.**

As a reminder: It is important that you consider both your feelings during each decision and the potential impact on your partner in the experiment. Your decisions can have a significant impact on their earnings. So, carefully weigh the pros and cons of each decision.

Take your time! You will make many decisions, and each has its advantages and disadvantages. Try to imagine what the other person would do in your place and how you would feel about the outcome.
